# PRC1 contributes to tumorigenesis of lung adenocarcinoma in association with the Wnt/β-catenin signaling pathway

**DOI:** 10.1186/s12943-017-0682-z

**Published:** 2017-06-24

**Authors:** Ping Zhan, Bin Zhang, Guang-min Xi, Ying Wu, Hong-bing Liu, Ya-fang Liu, Wu-jian Xu, Qing-qing Zhu, Feng Cai, Ze-jun Zhou, Ying-ying Miu, Xiao-xia Wang, Jia-jia Jin, Qian Li, Li-ping Qian, Tang-feng Lv, Yong Song

**Affiliations:** 1 Department of Respiratory Medicine, Jinling Hospital, Nanjing University School of Medicine, Nanjing, 210002 China; 2 Nanjing University Institute of Respiratory Medicine, Nanjing, 210002 China; 3 Department of Respiratory Medicine, Nanjing Chest Hospital, Medical School of Southeast University, Nanjing, 210029 China; 4 Department of Gastroenterology, Drum Tower Hospital, Medical School of Nanjing University, Nanjing, 210008 China; 5 Centre for Experimental Animal, Drum Tower Hospital, Medical School of Nanjing University, Nanjing, 210008 China

**Keywords:** PRC1, Lung adenocarcinoma, Prognosis, Proliferation, Wnt/β-catenin signaling

## Abstract

**Background:**

Protein regulator of cytokinesis-1 (PRC1) belongs to the microtubule-associated proteins (MAPs) family, and is involved in cytokinesis. Recent investigations suggest PRC1 involvement in human carcinogenesis, including breast carcinoma, hepatocellular carcinoma and etc. However, whether PRC1 contributes to lung adenocarcinoma tumorigenesis remains unknown.

**Methods:**

Quantitative reverse-transcription polymerase chain reaction (qRT-PCR), Western blotting and Immunohistochemical staining (IHC) were used to evaluate and contrast the PRC1 expression profile in lung adenocarcinoma and adjacent normal lung tissues. We examined the clinical use of PRC1 in lung adenocarcinoma prognosis. Additionally, the tumorigenesis impact of PRC1 in lung adenocarcinoma cells was verified via in vitro and in vivo metastasis and tumorigenesis assays. Notably, Next Generation Sequencing (NGS) was performed to investigate the molecular mechanism underlying the oncogenic role of PRC1 in lung adenocarcinoma.

**Results:**

PRC1 mRNA and protein expressions were upregulated in lung adenocarcinoma tissues compared to adjacent normal lung tissues. PRC1 protein overexpression correlated with lymph node metastasis and was an independent poor prognostic factor for lung adenocarcinoma patients. Our data implied that PRC1 depletion limited the proliferation and invasion of lung adenocarcinoma cells in vitro and lowered tumor development and lung metastasis in vivo. Remarkably, limiting PRC1 substantially prompted G2/M phase cell cycle arrest and apoptosis. Mechanistically, by conducting NGS on PRC1-depleted A549 cells and control cells, we discovered that PRC1 expression was significantly correlated with the Wnt signaling pathway.

**Conclusions:**

This investigation offers confirmation that PRC1 is a prognostic and promising therapeutic biomarker for people with lung adenocarcinoma and takes on a key part in the activation of the Wnt/β-catenin pathway in lung adenocarcinoma development.

**Electronic supplementary material:**

The online version of this article (doi:10.1186/s12943-017-0682-z) contains supplementary material, which is available to authorized users.

## Background

Lung cancer is the most frequently diagnosed cancer and the prominent reason for tumor-related death across the globe. Approximately 21.6% of all cancer-related deaths in China in 2015 were because of lung cancer [1]. Non-small cell lung cancer (NSCLC) and small cell lung cancer (SCLC) are responsible for an estimated 80–85% and 15–20% of all lung cancers, respectively. NSCLC can be categorized into two typical subtypes: adenocarcinoma (AD) and squamous cell carcinoma (SCC). Lung AD is the most common type and is responsible for approximately 40% of all lung cancers, while lung SCC is responsible for approximately 25% of all lung cancers [2]. While treatment boosts patient prognosis, the 5-year survival rate of advanced lung cancer patients is just 10–15% [3]. The poor prognosis of NSCLC is primarily because of the escalated rates of distant metastasis after resection. Therefore, determination of novel functional genes and biomarkers in tumor progression could offer different ways to treat patients with lung cancer.

Cytokinesis is the final stage of the mitosis. During cytokinesis, cell contents are divided into two independent daughter cells [4]_._ The protein regulator of cytokinesis-1 (PRC1, also known as Ase1/MAP65) gene, characterized as mitotic spindle associated cyclin dependent kinases(CDKs) substrate, was identified by Jiang et al. in 1998 [5]. The PRC1 gene is located at 15q26.1, and encodes a protein of 620 amino acids with a molecular size of 71 kDa [5]. Jiang et al. indicated that knockdown of endogenous PRC1 by siRNAs induced the dysregulation of mitosis and inhibited the process of cytokinesis, resulting in cells with two nuclei [5]. These results suggest that PRC1 is a CDK substrate and is involved in the process of cytokinesis [5]. In addition, accumulating evidence also suggests that PRC1 plays key roles in microtubule crosslinking [6, 7].

It has already been documented that PRC1 is overexpressed in different cancers, such as breast cancer [8], bladder cancer [9], hepatocellular carcinoma [10, 11], and pancreatic cancer [12]. An in vitro study showed that knockdown of PRC1 by using siRNA significantly inhibited the proliferation of breast and bladder cancer cells [8, 9]. Recently, Chen et al. found that PRC1 had an oncogenic function in promoting cancer proliferation, metastasis and tumorigenesis of hepatocellular carcinoma through a positive feedback loop of the Wnt signaling pathway [11]. Our previous study [13] firstly identified that PRC1 overexpression contributed to gastric cancer progression and can be targeted by piperlongumine via a p53-independent mechanism. These discoveries imply that PRC1 could take on crucial roles in tumorigenesis and could be a potential target for creating anticancer therapeutics for human cancer; however, as of right now, the expression profile of PRC1 in lung adenocarcinoma and its possible oncogenic role and molecular mechanisms have not been fully explained.

In this investigation, we limited our scope to the oncogenic role and molecular mechanism of PRC1 in lung adenocarcinoma. First, PRC1’s expression profile was evaluated in lung adenocarcinoma tissues and contrasted with nearby healthy lung tissues. We then examined the clinical use of PRC1 in the prognosis of lung adenocarcinoma. We further verified that the tumorigenesis impact of PRC1 in lung adenocarcinoma cells was by the induction of G2/M phase cell cycle arrest and apoptosis. Notably, Next Generation Sequencing (NGS) revealed that PRC1 was involved in the Wnt/β-catenin signaling pathway of lung adenocarcinoma tumorigenesis. Therefore, our results suggest that increased PRC1 expression may play an important role in lung adenocarcinoma initiation and progression.

## Methods

### Patients and tissue samples

Matched NSCLC and adjacent normal lung tissue were acquired from 30 patients who had primary surgical resection in Jinling Hospital, Nanjing University School of Medicine, China, between March and November 2013. Clinicopathological qualities of the NSCLC patients are provided in Additional file 1: Table S1.

### Immunohistochemical staining and scoring in tissue microarrays (TMAs)

Commercial TMAs (HLug-Ade180Sur-01, Shanghai Outdo Biotech, Shanghai, China) of 90 lung adenocarcinoma patients and matched adjacent normal lung tissue were employed to examine PRC1 expression. Antigen retrieval was conducted with high pressure for 5 min, with citrate buffer, pH 6.0. The sections were incubated overnight with the primary antibodies at 4 °C (rabbit monoclonal anti-PRC1 antibody, ab51248, Abcam). PBS was substituted for the primary antibody in the negative controls. Then, the slides were examined independently by 2 pathologists who were blinded to the patients’ clinical data. The staining scores for protein expression were done as we detailed prior [14]. An overall protein expression score (with an overall score ranging from 0 to 12) was determined by multiplying the intensity and positivity scores.

### Cell culture

The Institute of Biochemistry and Cell Biology of the Chinese Academy of Sciences (Shanghai, China) provided 3 lung adenocarcinoma cell lines (A549, SPC-A1, and NCI-H1299), 2 lung squamous carcinomas cell lines (NCI-H1703), and a human bronchial epithelial cell line (HBE). Every one of the cell lines was cultured as detailed prior [14].

### RNA extraction and qPCR assays

Total RNA extraction, reversed transcription and Quantitative reverse-transcription polymerase chain reaction (qRT-PCR) were performed as our previously described [14]. All of the reactions were performed in duplicate. The primer sequences are listed in Additional file 1: Table S2.

### Western blotting

Protein extraction and concentration measurement were determined according to the manufacturer’s instructions. Protein lysates were isolated by SDS-PAGE, moved to a PVDF membrane, and immunoblotted with antibodies as noted. The detailed methods have been described previously [14]. Information on the antibodies used is provided in the Additional file 1: Table S3.

### Plasmids

PRC1 expression vector (pcDNA3.1-PRC1) and β-catenin expression vector constructed by Genechem. (Shanghai, China), empty vector (pcDNA3.1) were purchased from Addgene.

### Lentivirus production

Lentivirus expressing shPRC1#1, shPRC1#2, or shControl were generated by GenePharma (Shanghai, China). The target sequences of these shRNA are listed in Additional file 1: Table S2. Viruses were condensed with the PEG-it virus precipitation solution (System Biosciences) and kept at −80 °C. For virus transduction, cells were plated 24 h prior to infection so that cells were approximately 50–60% confluent at the time of transduction. Cells were transduced with the appropriated amount of lentivirus for 24 h in the presence of polybrene (5 μg/mL). The media was then replaced with fresh medium. Cells were gathered for use in the experiments after culturing them for 4–6 days. Every one of the transfections was conducted based on the manufacturer’s instructions.

### Cell proliferation assays and colony formation assay

Cell proliferation was tracked with a Cell Proliferation Reagent Kit I (MTT) (Roche Applied Science, Switzerland). Colony formation assay was performed on a 6-well plate for 14 days; the colonies were set with methanol and stained with crystal violet. The observable colonies were manually tallied. Every one of the experiments was conducted as detailed prior [14]. Triplicate wells were evaluated for every treatment group.

### Cell migration/invasion assay

Transwell assays were completed with polycarbonate transwell filters (Corning Costar Corp., Cambridge, MA, USA) on the lower chambers, which were stocked with culture medium containing 10% FBS. Cells were treated for 24 h with shPRC1, and transfected shControl was suspended in RPMI1640 or DMEM medium and seeded in the upper chamber. After 24 h of culturing at 37 °C, we took out the upper portion of the cells prior to viewing, and the cells on the bottom were set in paraformaldehyde and stained with 0.1% crystal violet (Sigma). The cells on the bottom of the filter were tallied in 5 casually chosen fields and photographed. There were 3 independent filters that were evaluated for every experiment.

### Flow-cytometric analysis of apoptosis and the cell cycle

Cells transfected with shPRC1 and shControl were harvested 5 days after transfection by trypsinization. Cell cycle and apoptosis assay were performed by flow cytometry (BD Biosciences) as previously described [14]. All experiments were done in triplicate and repeated three independent times.

### Tumor formation assay in nude mice

Male athymic BALB/c nude mice (4 weeks old) were used for the tumor formation assay. The animal care and experimental protocols were approved by Nanjing University and were carried out in strict accordance with the Institutional Animal Care and Use guidelines. A549 cells stably transfected with lv-shPRC1 or empty vector were cultured for 4 days. Then, the cells were washed with phosphate-buffered saline and resuspended at 3 × 10^7^ cells/ml. A total of 200 μl of suspended cells transfected with control or shPRC1–1 was subcutaneously injected into the right and left side of the posterior flank of each mouse. Tumor volumes and weights were measured beginning from day 3 after the tumor cell injection. At 21 days post injection, the subcutaneous growth of each tumor was measured. Tumor volumes were calculated as length × width^2^ × 0.5. All surgeries were performed under sodium pentobarbital anesthesia, and every effort was made to minimize animal suffering [15].

### Tail vein injections into athymic mice

A549 cells that were steadily transfected with shPRC1 or the empty vector were gathered. Suspended cells (200 μl) were administered into the tail veins of 14 mice (4 weeks of age) that were killed at 7 weeks post-injection. The lungs were taken out and observable tumors on the surface of the lung were tallied and utilized in the additional analysis.

### Statistical analysis

Every one of the statistical evaluations was completed with GraphPad Prism 6.0 (GraphPad Software, La Jolla, California, USA). Data are expressed as the means ± SEM of at least three separate experiments. Statistically significant differences between the experimental and control groups were identified by Student’s *t* test. The immunohistochemistry results were analyzed using the Chi-squared test and Spearman’s rank correlation. Overall survival curves were determined based on the Kaplan–Meier procedure. *P* < 0.05 was deemed to be statistically significant.

## Results

### PRC1 is upregulated in NSCLC tissues

In an effort to determine the expression profile of PRC1 in NSCLC, the publicly accessible database Oncomine was employed to differentially examine PRC1 mRNA expression between NSCLC and normal pulmonary samples. We performed expression analysis of PRC1 with 4 microarray datasets from the Hou [16], Selamat [17], Su [18], and Landi [19] lung cancer groups that were downloaded from Oncomine. The expression of PRC1 mRNA was significantly escalated in NSCLC tissues in contrast to healthy tissues in these groups (Additional file 2: Figure S1A). Further, we examined the prognostic effect of PRC1 mRNA expression by Kaplan–Meier plotter analysis (http://www.kmplot.com) in the publicly accessible database. Kaplan–Meier plots of overall survival (Additional file 2: Figure S1B) implied that increased PRC1 mRNA expression is linked with lower survival in NSCLC. When stratified by various histological types, the patients with increased PRC1 mRNA expression had lower overall survival (OS) rates with NSCLC and lung adenocarcinoma, but not with lung squamous carcinoma.

**Fig. 1:**
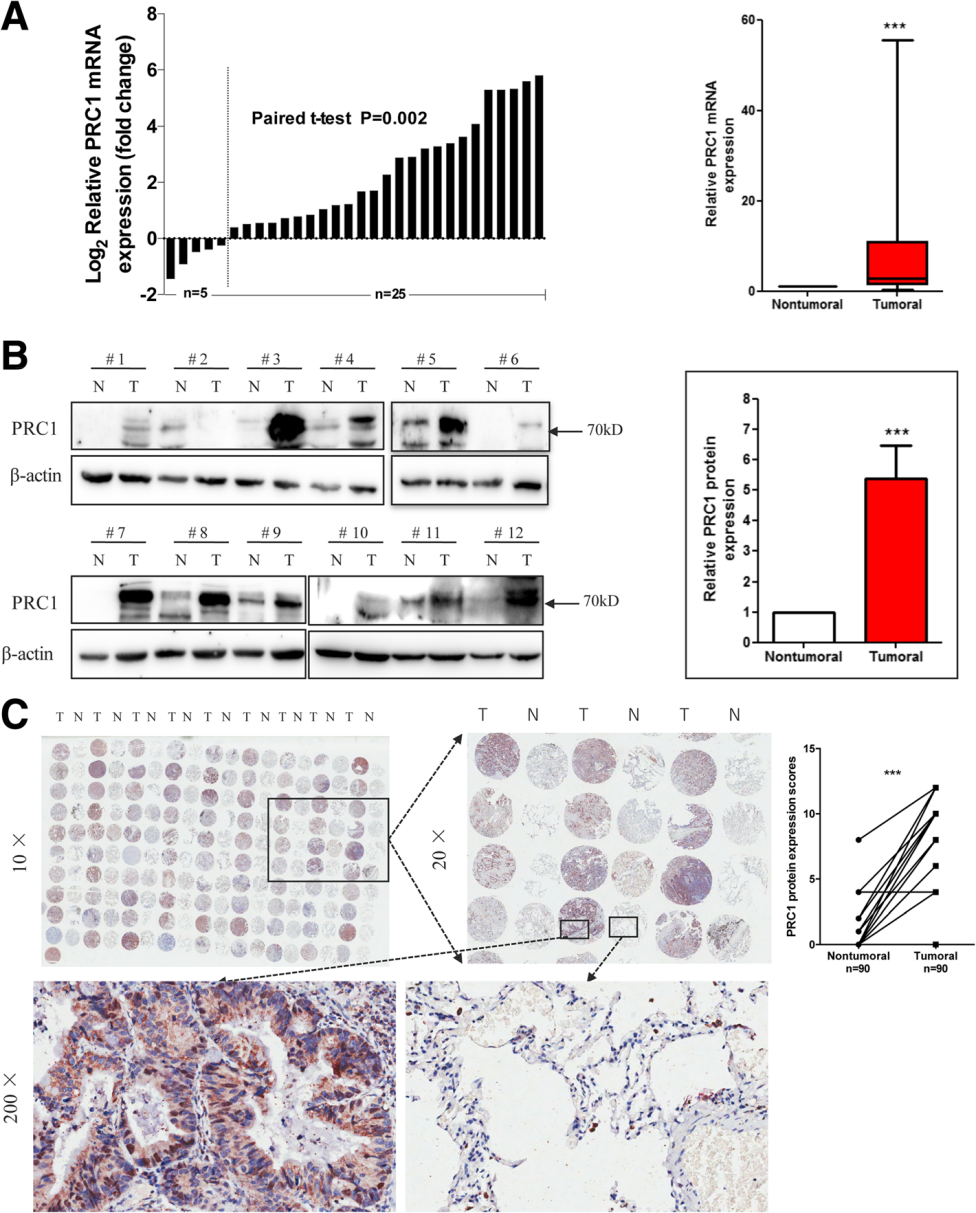
PRC1 is upregulated in primary lung adenocarcinoma tissues. **a** Real-time RT-PCR analysis of PRC1 expression levels in lung adenocarcinoma (AD) tissues and paired adjacent non-tumoral tissues (*n* = 30). The mRNA levels of PRC1 in lung AD tissues are significantly higher than those in non-tumoral tissues. ***P < 0.005 (N, non-tumoral; T, tumor). **b** Expression levels of PRC1 protein in 12 paired primary lung AD tissues were determined by western blotting. Quantitative analysis of relative expression density is shown in the *right* panel. Each column represents the mean ± standard deviation (SD); ***P < 0.005 (N, non-tumoral; T, tumor). **c** Expression levels of PRC1 protein in TMAs (90 paired lung AD tissues and paired adjacent non-tumoral tissues) were determined by immunohistochemistry (IHC). Representative examples of PRC1 staining in lung cancer tissues and non-tumoral lung tissues are shown. Statistical analysis of PRC1 scores is shown in the *right* panel. ***P < 0.005

Then, a total of 30 matched clinical NSCLC tissues and adjacent normal tissues were examined for PRC1 mRNA expression with qRT-PCR, and PRC1 expression was significantly upregulated in the cancerous tissues (*P* < 0.005; Fig. 1a). Further, Western blot assessment demonstrated heightened levels of PRC1 protein in the majority of the 12 matched NSCLC tissues in contrast to the nearby healthy tissues (*P* < 0.005; Fig. 1b).

Moreover, we evaluated the protein expression of PRC1 in clinical specimens via immunohistochemical (IHC) investigation of 90 pairs of lung adenocarcinoma carcinoma and matched adjacent normal tissues. PRC1 protein expression was mainly contained in the cytoplasm. Based on the stained area, the PRC1 protein expression score (the overall score ranged from 0 to 12) was determined by multiplying the intensity and positivity scores. The protein expression level of PRC1 in lung adenocarcinoma tissues was significantly greater than those in the corresponding nearby healthy tissues (*P* < 0.005) (Fig. 1c). The data suggest that PRC1 is overexpressed in human NSCLC, implying that PRC1 may have a part in cancer progression.

### PRC1 contributes to poor prognosis in lung adenocarcinoma patients

**Fig. 2:**
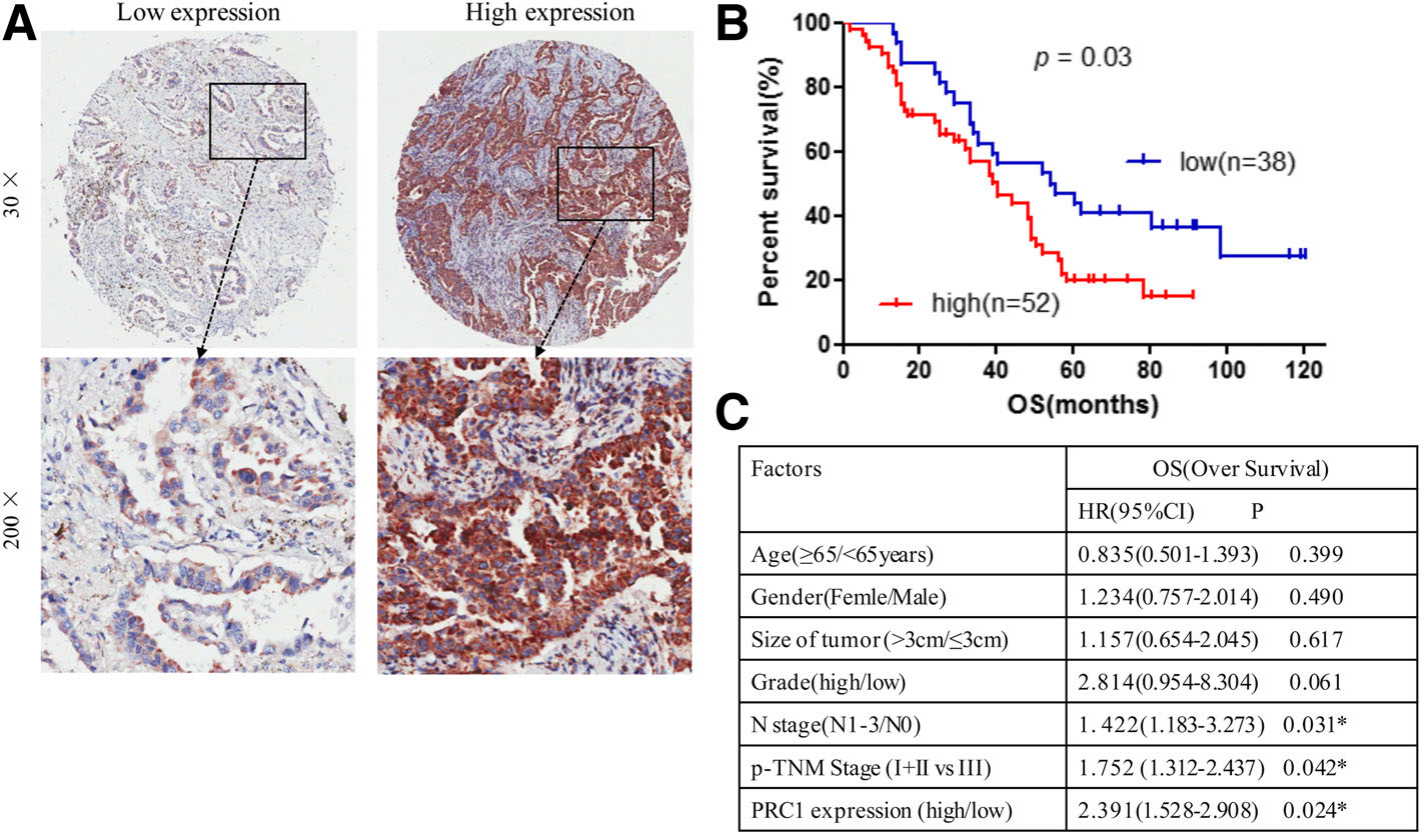
PRC1 overexpression predicted an unfavorable prognosis in patients with lung AD. **a** IHC staining of low expression (*left*) and high expression (*right*) of PRC1 in lung AD tissues with different magnification is shown. **b** Kaplan–Meier curves showing the overall survival rate of 90 patients with lung AD according to the expression status of PRC1 in the *lower* panel. The median staining score served as a cutoff to divide patients into high and low PRC1 expression levels. Red line: patients with high PRC1 expression, *blue line*: patients with low PRC1 expression. High PRC1 protein expression was associated with significantly decreased overall survival (*P* = 0.03, log-rank test). **c** Multivariate Cox regression analyses PRC1 for OS of patients in the study cohort (*n* = 90)

To determine the significance of PRC1 overexpression in predicting lung adenocarcinoma clinical outcome, 90 patients were divided into 2 groups according to the mean expression level of PRC1 (staining scores) by IHC (Fig. 2a). A PRC1 expression score ≤ 8.0 was defined as low expression and >8.0 was considered high expression. The correlation between PRC1 overexpression and clinicopathological factors of lung adenocarcinoma patients was assessed (Table 1). The statistical analyses showed that high expression of PRC1 protein also significantly correlated with lymph node metastasis (*P* = 0.041) and p-TNM stages (*P* = 0.039).

**Table 1:** Relationship of PRC1 protein expression with clinicopathological characteristics in lung adenocarcinoma patients

	Number of Patients	PRC1 protein expression		
		Low (≤8)	High(>8)	*P*
All patients	90	38	52	
Gender				0.533
Male	49	21	28	
Female	41	17	24	
Age				0.339
< 65 y. o.	51	23	28	
≥ 65 y. o.	39	15	24	
Size of tumor				0.499
≤ 3 cm	32	13	19	
> 3 cm	58	25	33	
Grade				0.184
Low	12	7	5	
High	78	31	47	
Lymph node metastasis (pN)				0.041*
N0	39	21	18	
N1–3	51	17	34	
p-TNM stages				0.039*
I	30	17	13	
II	21	5	16	
III	39	16	23	

^*^ *P* < 0.05

Interestingly, patients with higher levels of PRC1 protein had a shorter survival time than patients with lower PRC1 levels (*P* = 0.03, log-rank test, Fig. 2b). Notably, multivariate Cox regression analysis showed that N stage (HR = 1. 422, 95%CI = 1.183–3.273, *P* = 0.031), p-TNM Stage (HR = 1.752, 95% CI = 1.312–2.437, *P* = 0.042) and high expression of PRC1 protein (HR = 2.391, 95% CI = 1.528–2.908, *P* = 0.024) were independent prognostic factors for adenocarcinoma patients (Fig. 2c).

### Knockdown of PRC1 expression inhibits lung adenocarcinoma cells proliferation both in vitro and in vivo

**Fig. 3:**
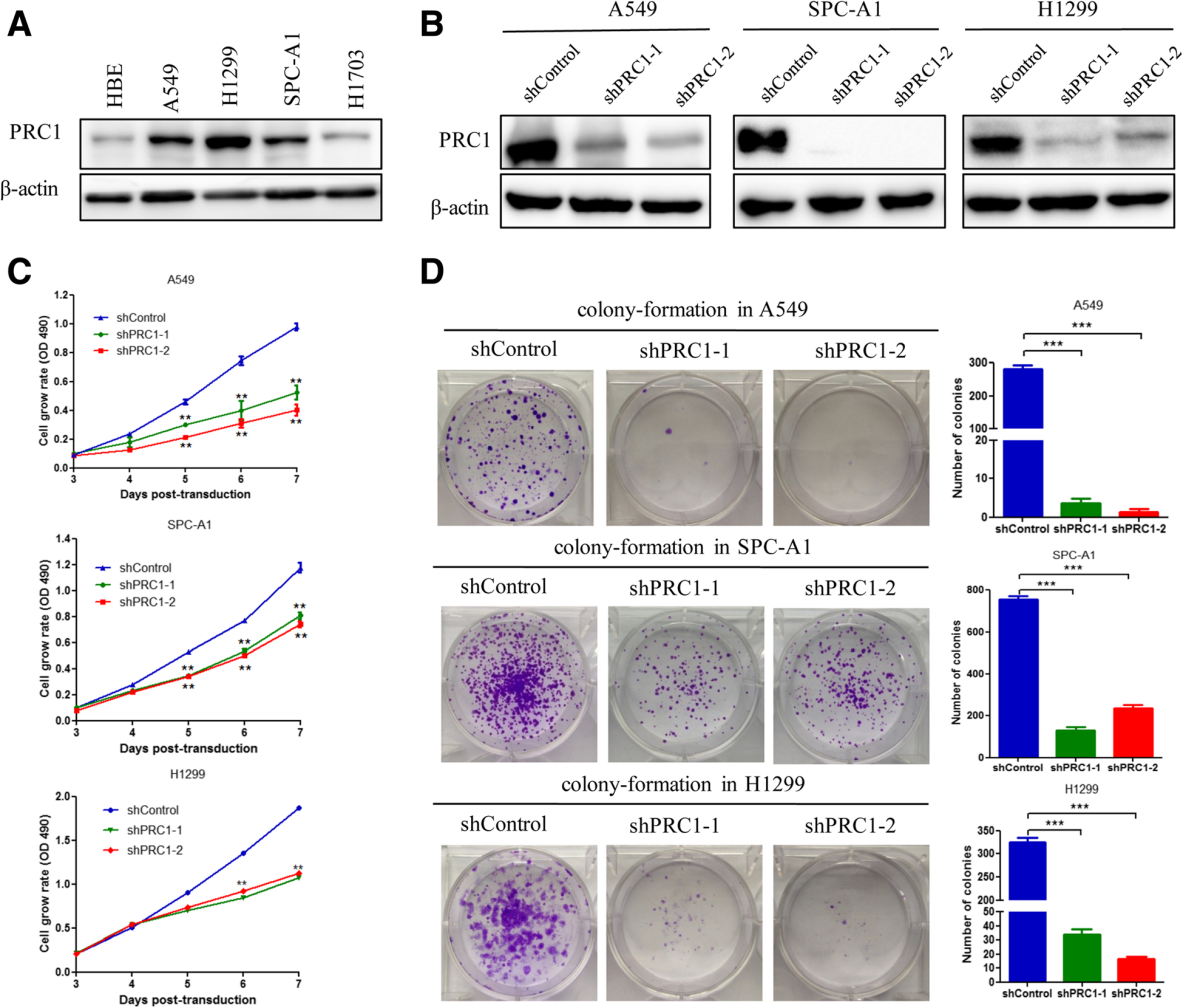
Effect of PRC1 gene silencing on the growth of lung adenocarcinoma in vitro*.* **a** PRC1 expression was detected by western blot analysis and qPCR in NSCLC cell lines (A549, SPC-A1, H1299, H1703, and SK-MES-1). β-actin was used as a loading control. **b** PRC1 expression was confirmed by qPCR and western blot analysis in lung adenocarcinoma cells (A549, SPC-A1, and H1299) transduced with lentiviruses expressing scramble (shControl) or PRC1-targeting (shPRC1–1 and shPRC1–2) shRNA. Cells were harvested 5 days after viral transduction. β-actin was used as a loading control. **c** MTT assay was performed to determine the proliferation of lentiviruses shPRC1 transfected A549, SPC-A1, and H1299 cells. **d** A colony formation assay of A549, SPC-A1, and H1299 cells transduced with lentiviruses expressing the indicated shRNA. Cells were grown for 10–14 days under the selection of puromycin (0.25 mg/ mL) and stained with crystal violet. The colonies were counted and captured. The data represent the mean ± S.D. from three independent experiments. **P < 0.01, ***P < 0.005

We next performed qRT-PCR and Western blot analysis to determine the expression level of PRC1 in 4 human NSCLC cell lines, which include one squamous carcinoma cell lines and 3 adenocarcinoma cell lines, and HBE We observed that PRC1 expression was elevated in three lung adenocarcinoma cell lines, whereas PRC1 expression was lower in one lung squamous carcinoma cell lines and HBE (Fig. 3a, Additional file 3: Figure S2A). To investigate the mechanism by which PRC1 contributes to the malignancy of lung adenocarcinoma, knock down of endogenous PRC1 was performed by lentivirus transfection of shPRC1 in A549, SPC-A1, and H1299 cells. Cells were harvested 5 days after viral transduction and then PRC1 expression levels were determined. The data demonstrated that shRNAs targeting PRC1 mRNA reduced its expression more than 60–80% relative to the negative control shRNA in A549, SPC-A1 and H1299 cells (Fig. 3b and Additional file 3: Figure S2B).

Next, we examined the effect of PRC1 knockdown on the proliferation of three lung adenocarcinoma cells. Knockdown of the PRC1 gene led to a significant decrease of cell viability in A549, SPC-A1 and H1299 cells as determined by MTT assay (Fig. 3c). Furthermore, PRC1 knockdown substantially reduced the number and size of colonies in A549, SPC-A1 and H1299 cells (Fig. 3d). These results suggest that PRC1 gene silencing inhibits lung adenocarcinoma cell proliferation in vitro*.*

**Fig. 4:**
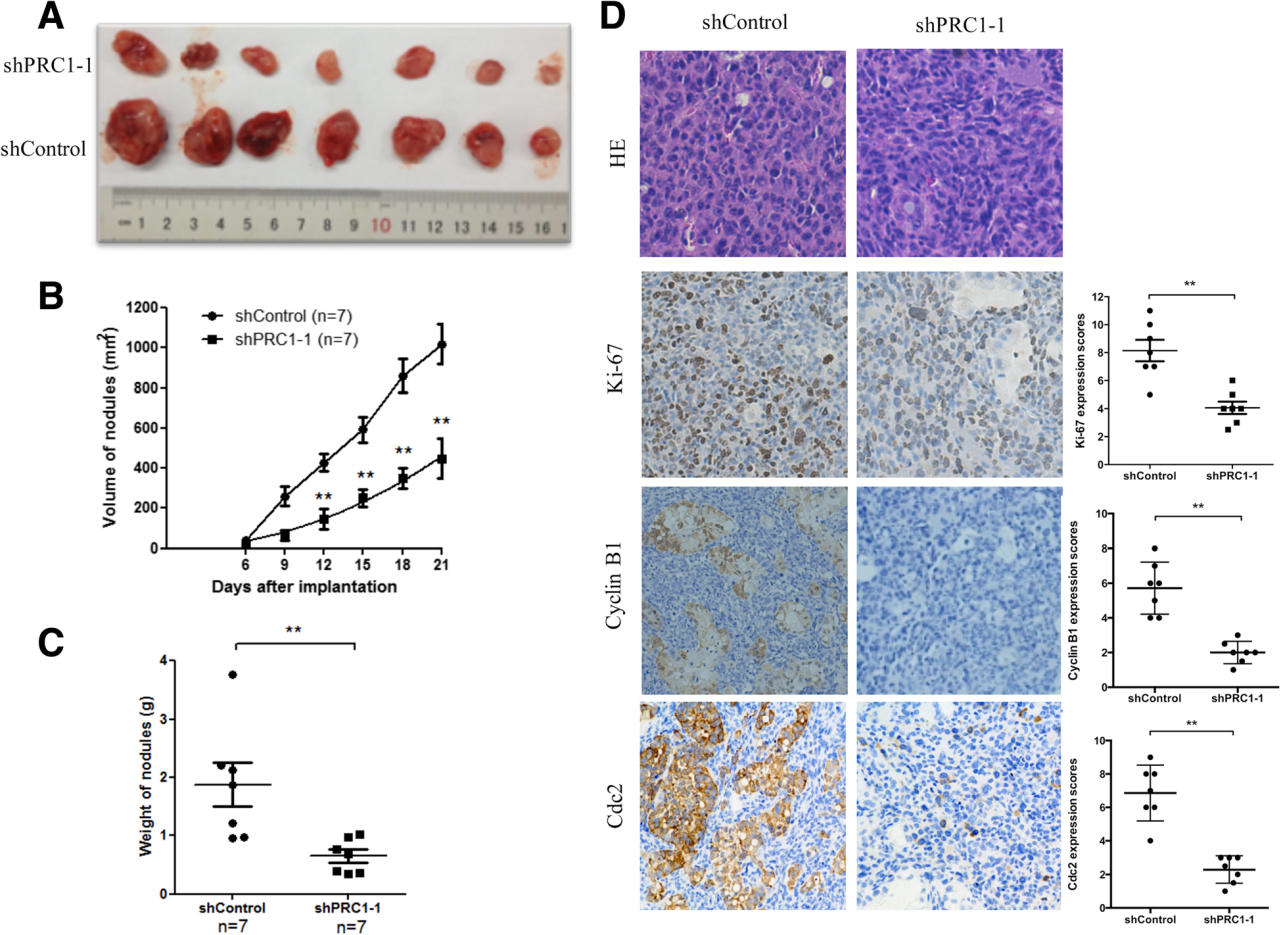
Downregulation of PRC1 inhibits the tumor growth of subcutaneous xenograft tumors in vivo*.* A549 cells were transduced with lentiviruses shPRC1–1 or shControl and then injected into mouse flanks as described in the text. Tumor growth was measured every 3 days after injection, and the tumors were harvested at day 21 and weighed. **a** The resected tumors from each group (*n* = 7) were photographed. **b** Tumor growth curves were summarized, and the error bars indicate the standard deviation (SD). **c** Tumor weight is represented as means mean ± S.D. when the tumors were harvested. **d** Representative images (200×) of HE staining of the resected tumor. IHC analysis showed that PRC1-knockdown decreased the proliferation index Ki67, and reduced the expression of cyclin B1 and Cdc2. *P < 0.05, **P < 0.01

To further explore the effect of PRC1 on tumor growth in vivo, 14 severe combined immunodeficient mice were subcutaneously injected with A549 cells transduced with shControl or shPRC1–1. Six days after injection, all mice developed detectable tumors. At 21 days after tumor inoculation, mice harboring tumors with reduced PRC1 expression showed dramatically decreased tumor growth compared to the control groups, as demonstrated by the substantially reduced tumor size and weight (Fig. 4a–c). The tumors were resected and analyzed by hematoxylin and eosin (HE) staining and IHC staining. Tumor tissues derived from shPRC1–1 transduced cells exhibited reduced positivity for Ki67, Cyclin B1 and Cdc2 compared with the control groups (Fig. 4d). Therefore, PRC1 down-regulation inhibits the growth of established lung adenocarcinoma in xenografts.

### Overexpression of PRC1 promotes on the proliferation in A549 and HBE cells

**Fig. 5:**
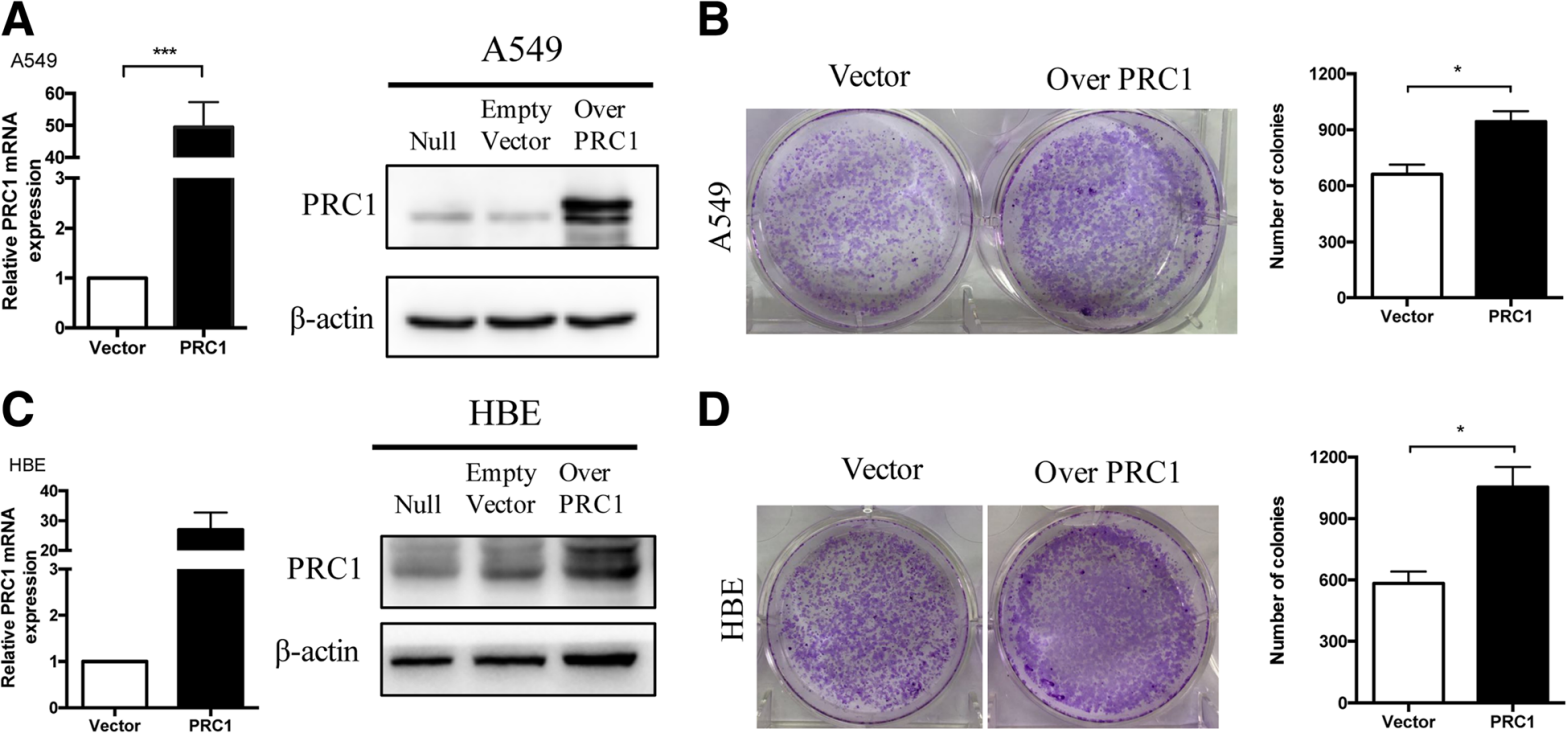
Overexpression of PRC1 promotes on the proliferation in A549 and HBE cells **a** qRT-PCR and Western blotting analysis of PRC1 mRNA and protein level in A549 cells transfected with PRC1 expressing plasmid or empty vector. β-Actin mRNA expression was used as an internal control. Experiment was conducted in triplicate. *** *P* < 0.005. **b** A colony formation assay of A549 cells transduced with PRC1 expressing plasmid or empty vector. The colonies were counted and captured. The data represent the mean ± S.D. from three independent experiments, *P < 0.05. **c** PRC1 mRNA and protein level in HBE cells transfected with PRC1 expressing plasmid or empty vector, *** *P* < 0.005. **d** A colony formation assay of HBE cells transduced with PRC1 expressing plasmid or empty vector, *P < 0.05

To further test whether overexpression of PRC1 regulates the cell proliferation in A549 and HBE cells, A549 and HBE cells were transfected with PRC1 expressing plasmid or empty vector. PRC1 mRNA and protein level in A549 and HBE cells were overexpressed accordingly (Fig. 5a, c). Furthermore, ectopic expression of PRC1 also promoted the number and size of colonies in A549 and HBE cells (Fig. 5b, d). Collectively, these data suggested that PRC1 was critical for lung adenocarcinoma cell proliferation in A549 and HBE cells.

### PRC1 knockdown suppresses lung adenocarcinoma metastasis both in vitro and in vivo

**Fig. 6:**
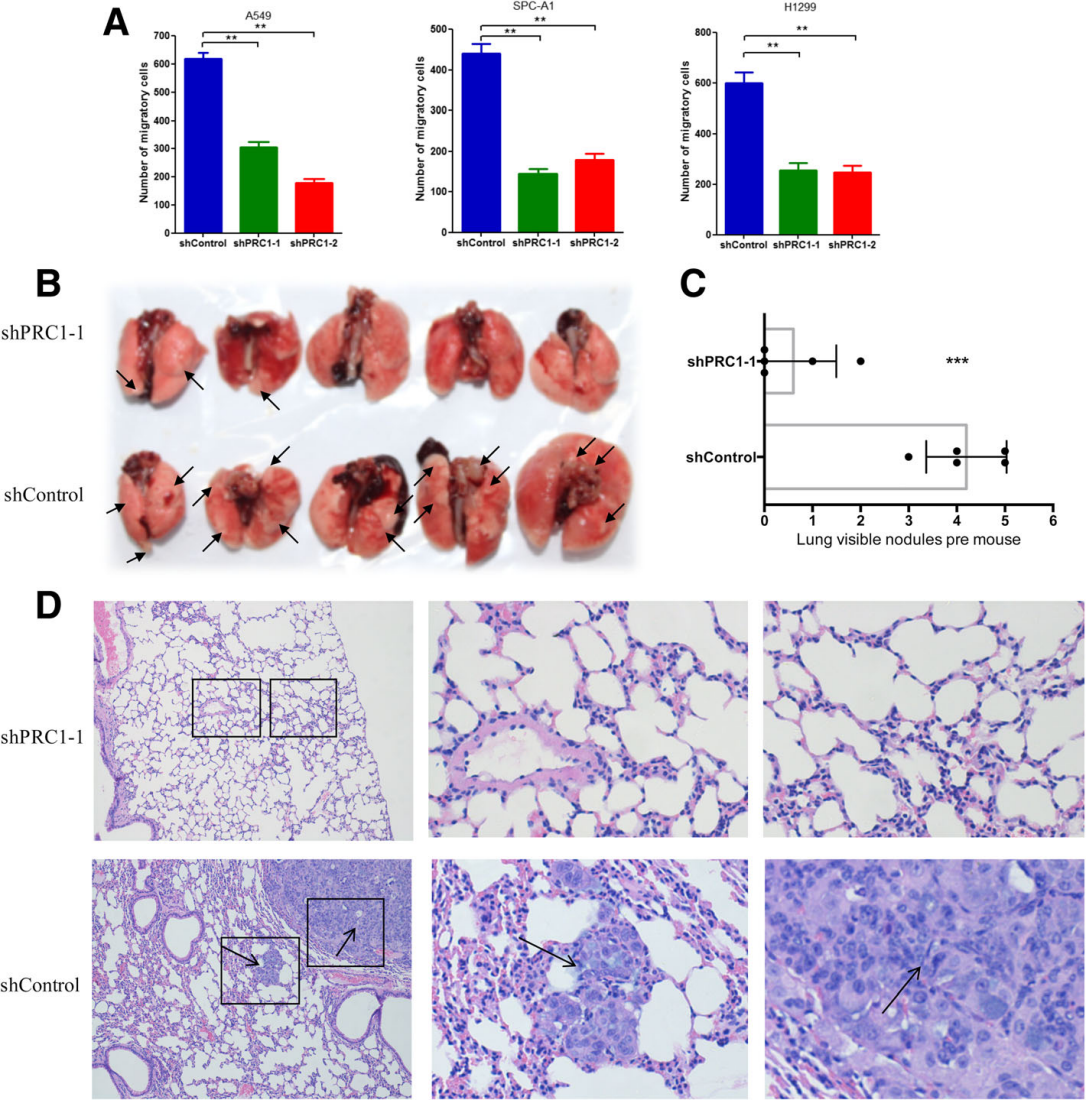
PRC1 knockdown significantly attenuates NSCLC cell migration in vitro and metastasis in vivo*.* **a** Transwell assays were performed to determine the migratory abilities of A549, SPC-A1, and H1299 cells transduced with lentiviruses expressing the indicated shRNA. **b** Analysis of an experimental metastasis animal model was performed by injecting A549 cells transduced with lentiviruses shPRC1–1 or shControl into nude mice via the tail vein. Representative lung images taken from mice at week 6 are shown. **c** the number of tumor nodules on lung surfaces are shown. **d** Representative HE staining of lung tissues from the different groups is shown. **P < 0.01, ***P < 0.005

We further investigated whether PRC1 regulates lung adenocarcinoma cells motility in vitro using transwell migration assays. As expected, knockdown of PRC1 significantly decreased cell migration through the membrane in A549, SPC-A1 and H1299 cells compared to the controls (Fig. 6a and Additional file 4: Figure S3). To examine if PRC1 promotes tumor metastasis in vivo, A549 cells transduced with lentivirus shRNA were injected into the tail vein of male nude mice. Knockdown of PRC1 led to a reduction in the number of metastatic nodules compared to the control group (Fig. 6b, c). In addition, HE staining of lung sections also demonstrated a significantly reduced number of metastatic nodules in the shPRC1-transduced tumors (Fig. 6d). These in vivo and in vitro results synergistically demonstrated that PRC1 plays an important regulatory role in lung adenocarcinoma metastasis.

### Depletion of PRC1 expression leads to G2/M cell cycle arrest and apoptosis in lung adenocarcinoma cells

**Fig. 7:**
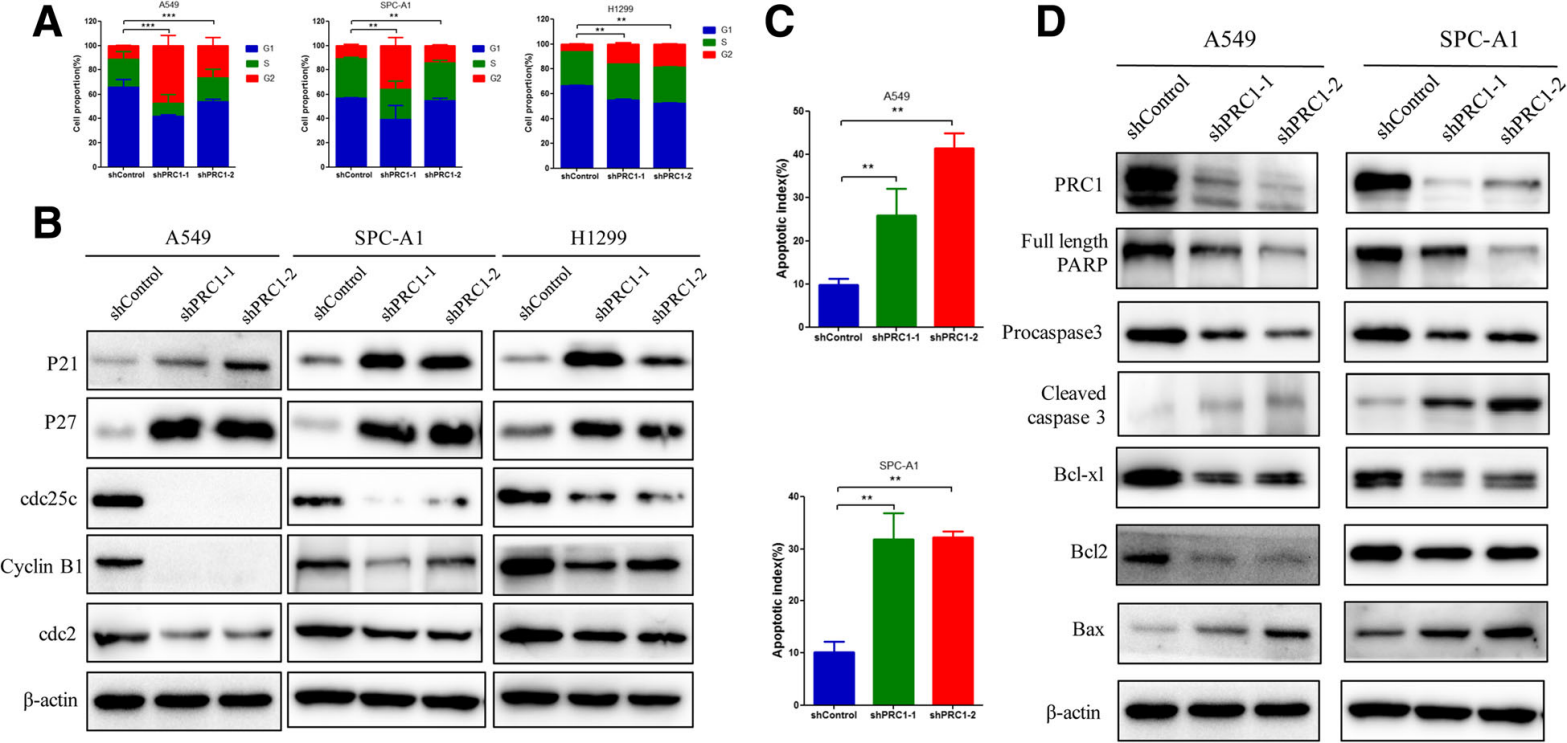
PRC1 knockdown induces G2/M phase arrest and apoptosis in vitro*.* **a** Cell-cycle analysis was determined in A549, SPC-A1, and H1299 cells transduced with shPRC1 or shControl. The DNA content was quantified by flow cytometric analysis. The percentage of cells in each population was shown as the mean ± S.D. from three independent experiments. Student’s t-test **P < 0.01, ***P < 0.005. **b** Western blot analysis of G2/M transition-related proteins in A549, SPC-A1, and H1299 cells transduced with shPRC1. Similar experiments were repeated at least twice with similar results. **c** Apoptosis in A549 and SPC-A1 cells transduced with shPRC1 was performed by flow cytometry and the apoptotic index was defined as the percentage of apoptotic cells. **P < 0.01. **d** Caspase-3, PARP, Bcl-xl, Bcl2, and Bax were evaluated by western blotting in both cell lines after treatment with shPRC1. Similar experiments were repeated at least twice with similar results

To investigate the role of PRC1 in the regulation of cell cycle progression in lung adenocarcinoma cells, flow cytometric analysis was performed on A549, SPC-A1 and H1299 cells transduced with shPRC1 or shControl. The results showed an accumulation of cells in the G2/M phase in three cell lines following PRC1 depletion (Fig. 7a and Additional file 5: Figure S4). Furthermore, western blot analysis of the key proteins associated with the G2/M phase [20, 21] showed there were upregulation of p21 and p27, and a decrease of cyclin B1, Cdc2 and Cdc25c (Fig. 7b). These data illustrated that silencing PRC1 expression could arrest cells at the G2/M phases by regulating the G2/M associated proteins. This suggests that PRC1 plays a role in lung adenocarcinoma cell development through the regulation of the cell cycle progression at the G2/M phase.

Interestingly, we found that A549 cells transduced with shPRC1 became shrunken and detached, or even floated. It is speculated that apoptosis may play a role in this observation (Additional file 6: Figure S5). We then used the Annexin V/PI assay to determine whether silencing PRC1 expression could induce apoptosis in lung adenocarcinoma cells. There was a significant increase in the apoptotic population in A549 and SPC-A1 cells transduced with shPRC1 (Fig. 7c and Additional file 7: Figure S6). At the molecular level, there was a reduction in the expresson of anti-apoptotic proteins such as full length PARP, procaspase3, Bcl2 and Bcl-xl. However, there was an induction of the pro-apoptotic protein cleaved caspase3 and Bax (Fig. 7d). These data added further evidence for the oncogenic role of PRC1 in lung adenocarcinoma cells through the regulation of the G2/M cell cycle and apoptosis.

### **PRC1 correlates with the** Wnt/β-catenin **pathway**

**Fig. 8:**
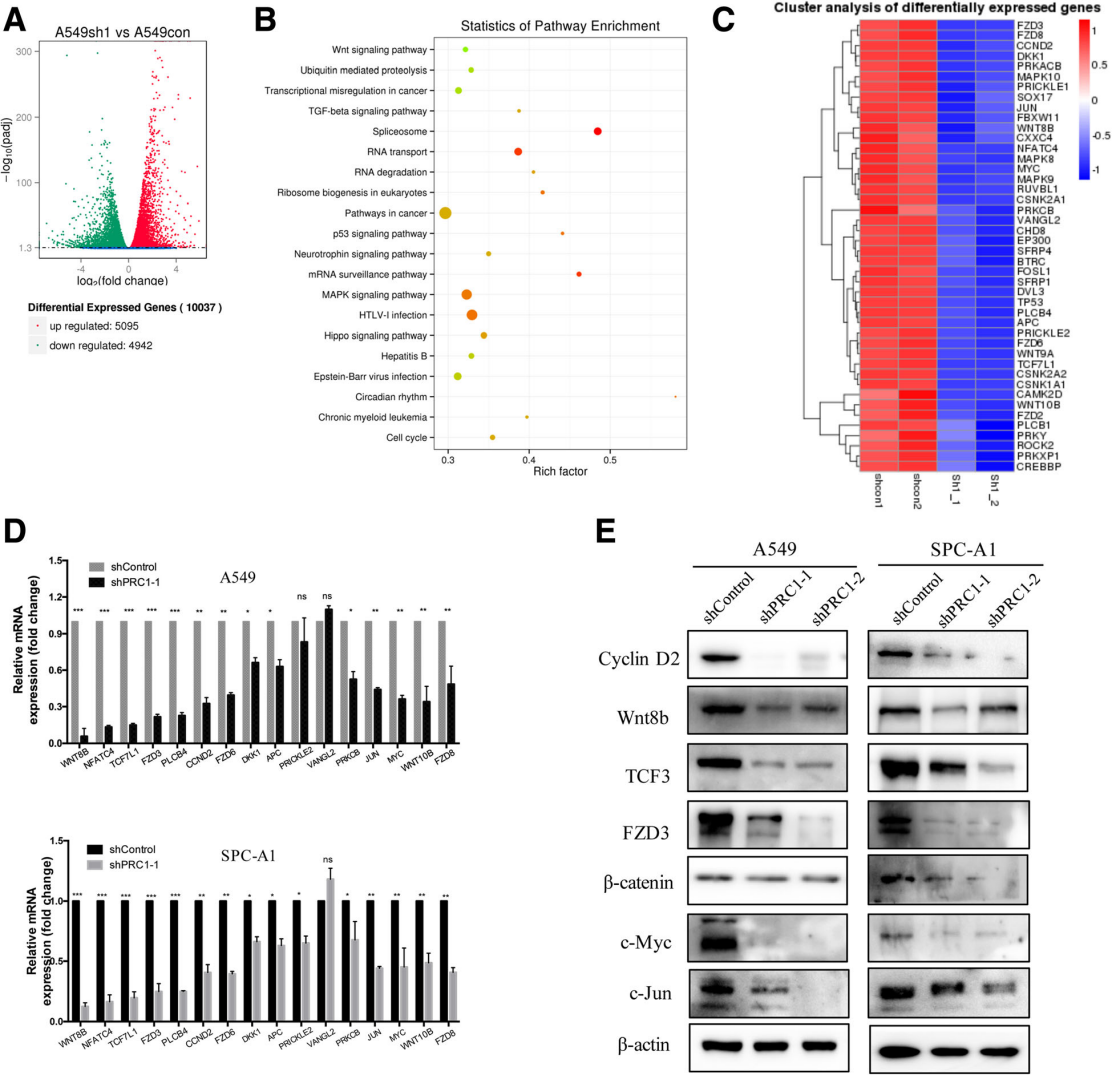
PRC1 correlated the WNT pathway through NGS analysis in vitro*.* **a** RNA transcriptome sequencing analysis was performed to analyze the gene expression profile of A549 cells transduced with shPRC1–1 or shControl. A volcano plot showed the differentially expressed genes. **b** KEGG pathway analysis for all genes with altered expression between the negative control group and shPRC1–1 knockdown group cells in vitro. **c** Expression heatmap of Wnt pathway genes significantly differentially expressed transcripts after the transduction of shPRC1–1. Expression profiles of the 44 probes in A549 cells with shControl (*left*) and shPRC1–1 (*right*) are shown. *Red* and *green* indicate up- and downregulation, respectively. **d** qRT-PCR analysis of the mRNA expression levels of 16 Wnt pathway relevant DEGs in shControl vs. shPRC1–1-treated A549 cells and SPC-A1. **e** Western blot analysis of the level of 16 Wnt pathway relevant DEGs in shControl vs. shPRC1–1-treated A549 cells and SPC-A1

To investigate the molecular mechanism underlying the oncogenic role of PRC1 in lung adenocarcinoma, we utilized NGS to assess the gene expression profiles of PRC1-depleted A549 cells and control cells. This unbiased genome-scale analysis identified 10,037 differentially expressed genes (DEGs) [log2(FoldChange)] > 1 and *P* < 0.05) in A549 cells after PRC1 knockdown compared to controls. This includes 4942 downregulated DEGs and 5096 upregulated DEGs (Fig. 8a). Furthermore, to investigate the functional processes that were affected by PRC1-mediated transcriptional regulation, KEGG (Kyoto Encyclopedia of Genes and Genomes) analysis was performed. The pathway analysis showed that the Wnt/β-catenin, TGF- β, Hippo, p53, MAPK and cell cycle pathways were significantly enriched from downregulated DEGs (Fig. 8b and c). The Wnt/β-catenin pathway was the most significantly dysregulated in response to PRC1-depeletion. The NGS and KEGG analysis suggests that the Wnt/β-catenin pathway might be involved in the affected functional processes in PRC1-depeleted cells, which was also consistent with the study of Chen et al. [11].

**Fig. 9:**
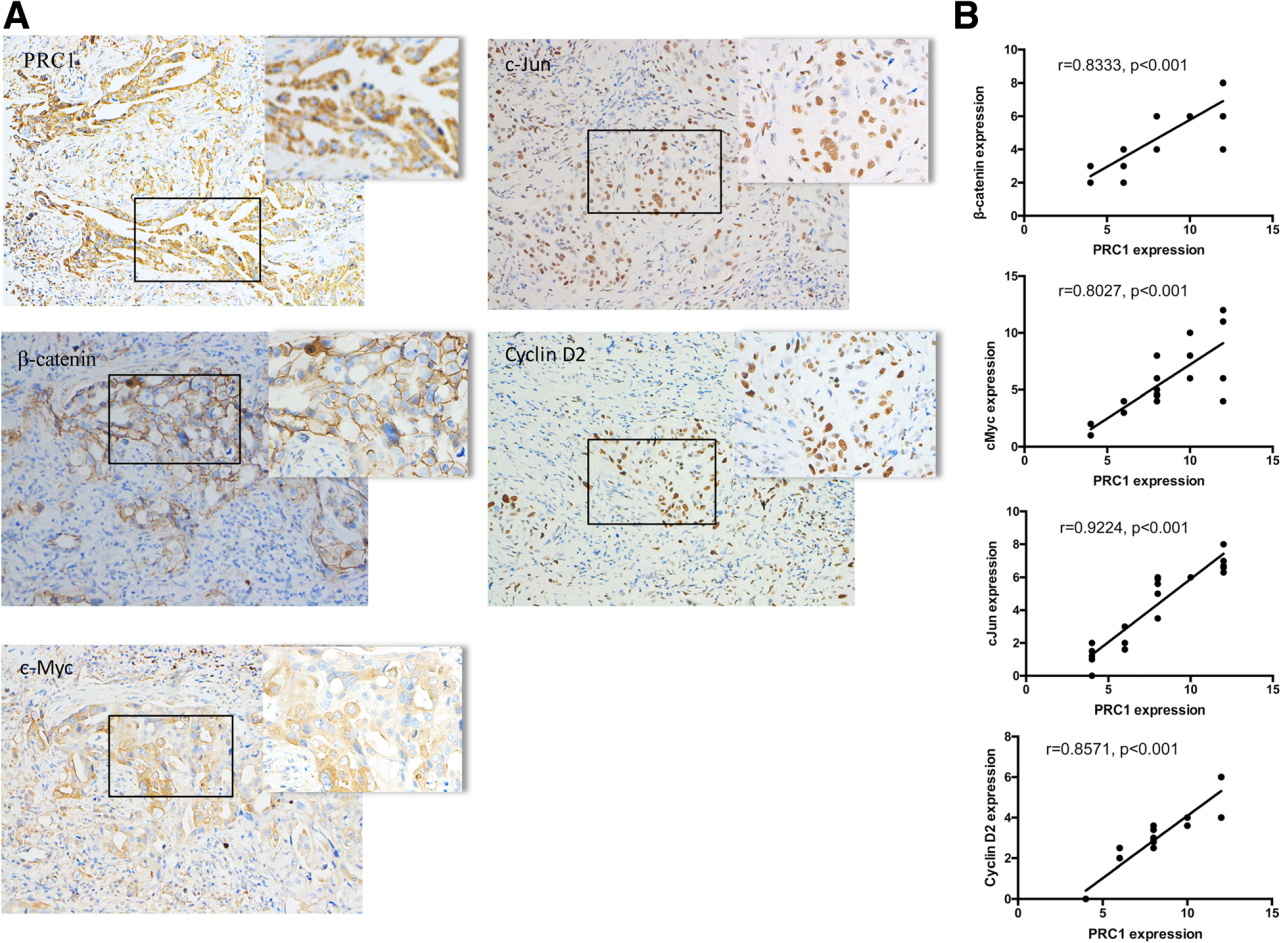
The relationship between PRC1 and Wnt pathway relevant genes in patients with lung adenocarcinoma. **a** IHC staining of PRC1, β-catenin, c-Myc, c-Jun, and Cyclin D2 expression in patient tissues (*n* = 25). **b** The correlation the PRC1 and β-catenin, c-Myc, c-Jun, and cyclin D2 based on IHC assays

We further validated relevant genes involved in the Wnt/β-catenin pathway in vitro and the correlation with PRC1 in vivo. An expression heatmap of 44 Wnt/β-catenin pathway relevant DEGs after the transduction of shPRC1–1 is shown in Fig. 8c. We next confirmed the reduction of several representative genes such as Wnt8b, TCF7L1, FZD3, PLCB4, Cyclin D2, c-Myc, c-Jun and etc. in A549 and SPC-A1 by qRT-PCR (Fig. 8d). Western blot analysis consistently showed that the protein expression levels of these genes in A549 and SPC-A1 cells were significantly reduced after transduction of shPRC1 (Fig. 8e). Moreover, β-catenin, c-Myc, c-Jun, Cyclin D2 and PRC1 protein expression were examined by IHC in tissue sections of 25 lung adenocarcinoma (Fig. 9a). In addition, the Spearman’s rank correlation analysis revealed that PRC1 was positively correlated with β-catenin, c-Myc, c-Jun and Cyclin D2, respectively (Fig. 9b), suggesting that PRC1 is associated with the Wnt/β-catenin signaling pathway in in vivo*.*

### β-catenin reverses the tumorigenic growth in the lentiviruses shPRC1 transfected A549

**Fig. 10:**
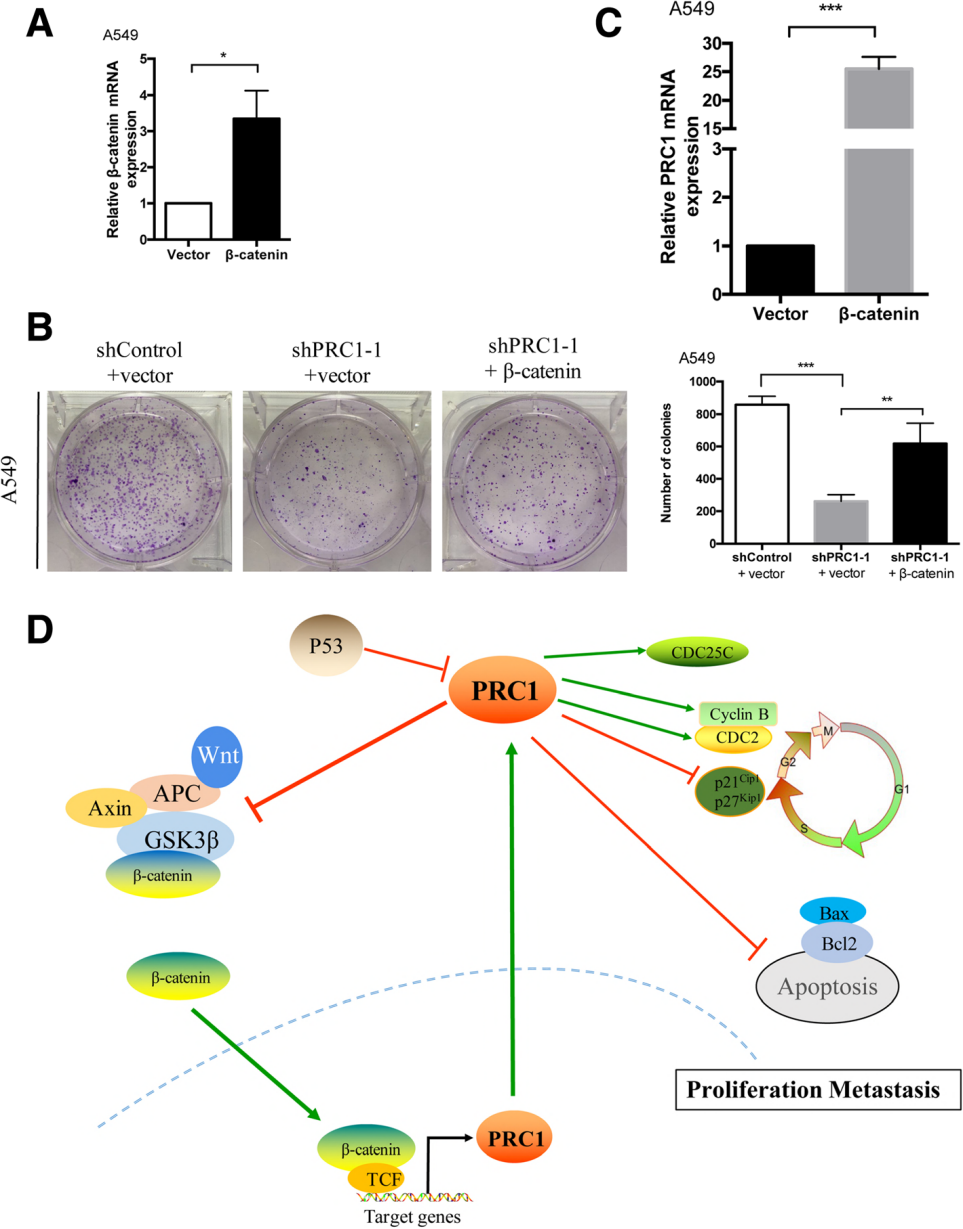
β-catenin reverses the tumorigenic growth in the lentiviruses shPRC1 transfected A549, and up-regulates the expression of PRC1. **a** qRT-PCR analysis of β-catenin mRNA level in A549 cells transfected with β-catenin expressing plasmid or empty vector. β-Actin mRNA expression was used as an internal control. Experiment was conducted in triplicate. * *P* < 0.05. **b** A colony formation assay of A549 cells stably transfected with control or PRC1 shRNA with or without β-catenin expressing plasmid. The colonies were counted and captured. *P < 0.05. **c** qRT-PCR analysis of PRC1 mRNA level in A549 cells transfected with β-catenin expressing plasmid or empty vector. **d** A proposed working model of PRC1 functions lung adenocarcinoma. PRC1 gene is directly suppressed by P53 and could promote the activation of Wnt/β-catenin signaling in a positive feedback regulatory network. Loss of PRC1 induces G2/M phase arrest and apoptosis in vitro, PRC1 expression is associated with high Wnt/β-catenin potency in lung adenocarcinoma patient tissues and promote metastasis and proliferation

We then investigated whether β-catenin reverses the tumorigenic growth in the lentiviruses shPRC1 transfected A549, A549 cells were transfected with β-catenin expressing plasmid or empty vector. β-catenin mRNA level in A549 cells were overexpressed accordingly (Fig. 10a). Furthermore, the colony formation assay indicated that β-catenin reverses the tumorigenic growth in the lentiviruses shPRC1 transfected A549 (Fig. 10b). Interestingly, β-catenin also up-regulated the expression of PRC1 remarkably (Fig. 10c). As previously described [11], these evidences indicated that PRC1 and Wnt/β-catenin signaling could be also a positive feedback regulatory network in lung adenocarcinoma.

Taken together, PRC1 gene is directly suppressed by P53 [22], and could promote the activation of downstream target genes by inhibiting the membrane sequestration of APC/β-catenin destruction complex, inducing the nuclear translocation of β-catenin [11]. More importantly, overexpression of β-catenin could increase the expression of PRC1 in a positive feedback loop. Additionally, loss of PRC1 induces G2/M phase arrest and apoptosis in vitro, PRC1 expression is associated with high Wnt/β-catenin potency in lung adenocarcinoma patient tissues and PRC1 protein overexpression was an independent poor prognostic factor for lung adenocarcinoma patients.

## Discussion

Cytokinesis is a complex process involving sophisticated biochemical and cellular dynamics factors. It has been shown that abnormal cytokinesis can lead to cell chromosome instability and malignant transformation [4, 23, 24]. As a critical protein of cytokinesis, dysregulated expression of PRC1 gives rise to disorder of cytokinesis. The previous studies [8, 9, 11, 13] have implicated that PRC1 as an oncogenic factor in tumorigenesis of breast, bladder cancer, hepatocellular carcinoma and gastric carcinoma. Herein, we demonstrated that both mRNA and protein expressions of PRC1 were upregulated in lung adenocarcinoma tissues compared with adjacent normal lung tissues. We further determined that overexpression of PRC1 protein correlates with lymph node metastasis and is an independent poor prognostic factor for lung adenocarcinoma patients.

Previous studies have suggested that p53 directly suppresses PRC1 gene transcription through interaction with the PRC1 gene promoter in breast cancer cells [22], indicating that PRC1 is a common downstream target of p53. There is sufficient evidence to demonstrate that p53, as a famous tumor-suppressor gene, can enhance the sensitivity of tumor cells to radiotherapy and chemotherapy, induce the cell cycle arrest and inhibit cell growth and proliferation of tumor cells [25–27]. By means of inducing the expression of the p53 gene, 5-fluorouracil, a chemotherapy drug, could inhibit tumor growth in hepatocellular carcinoma and colon cancer cells [27, 28]. In addition, PRC1 was also confirmed as a downstream target of heat shock transcription factor 2 (HSF2) and is involved in the regulation of cytokinesis [29]. Moreover, the present study demonstrates that reduced expression of PRC1 suppresses the proliferation and invasion of lung adenocarcinoma cells in vitro and in vivo. Collectively, these results suggest that silencing of PRC1 could inhibit tumorigenesis by blocking the cytokinesis in a variety of cancer cells, and further demonstrating that PRC1 is expected to become a new target for gene therapy of tumors.

It is common knowledge that metastasis of cancer cells is a complex, multi-step and continuous procedure that depends on histologic types, intracellular environment and the interactions between cells and extracellular matrix. Metastasis also involves complex networks between different signaling pathways and different genes [30]. Not long ago, Chen et al. [11] showed that the expression and distribution of PRC1 is drastically controlled by Wnt3a signaling. Knockdown of PRC1 inhibited the transcriptional activity of transcription factor (TCF), and reduced Wnt target expression of nuclear β-catenin levels in hepatocellular carcinoma. Consistent with this finding, our results also confirmed that PRC1 is involved in the regulation of the Wnt/β-catenin signaling pathway in lung adenocarcinoma. To additionally verify the interaction between PRC1 and Wnt/β-catenin pathway relevant genes, we contrasted the protein expression patterns in tumor specimens obtained from patients with lung adenocarcinoma. In fact, those with PRC1 overexpression demonstrated coordinated overexpression of β-catenin, c-Myc, c-Jun, and cyclin D2, while others with reduced PRC1 expression revealed a diminished expression of β-catenin, c-Myc, c-Jun, and cyclin D2.

Wnt/β-catenin signaling is tightly regulated at multiple cellular levels and is dysregulated in lung cancer [31]. Our prior meta-analysis [32] demonstrated that the overexpression of Wnt proteins (Wnt1 and Wnt5a) was notably linked to unfavorable overall survival in lung cancer patients. Chen et al. [11] discovered that PRC1 interacts with the β-catenin destruction complex, controls Wnt3a-induced membrane sequestration of the damaging complex, limits adenomatous polyposis coli (APC) durability, and encourages β-catenin release from the APC complex. In the present study, the Nest Generation Sequencing (NGS) and bioinformatics analysis showed that after knocking down PRC1, differentially expressed genes (DEGs) were enriched in the Wnt/β-catenin, TGF- β, Hippo, p53, MAPK and cell cycle pathways. Of these pathways, the Wnt/β-catenin pathway was the most significantly dysregulated in response to PRC1-depeletion based on NGS analysis. In addition, Wnt/β-catenin pathway relevant genes were further validated at the mRNA and protein expression levels in vitro, and found that Wnt8b was the most significantly downregulated gene. However, the precise molecular mechanisms underlying the role of the Wnt/β-catenin signaling pathway in the regulation of PRC1 in lung adenocarcinoma remain unclear and will be investigated in future studies.

## Conclusions

In summary, we determined for the first time the expression pattern and molecular mechanism of PRC1 in lung adenocarcinoma. Our results provide a basis for the concept that overexpression of PRC1 in human lung adenocarcinoma may be important in the acquisition of an aggressive and poor prognostic phenotype. Moreover, reduced expression of PRC1 suppresses the proliferation and invasion of lung adenocarcinoma cells in vitro and in vivo by inducing G2/M phase cell cycle arrest and apoptosis, suggesting that PRC1 may serve as an oncogene and potential therapeutic biomarker for lung adenocarcinoma. The preliminary mechanistic studies indicate that PRC1 may play a critical role in the control of lung adenocarcinoma aggressiveness by activating the PRC1/Wnt/β-catenin feedback loop.

## Additional files

Additional file 1: Table S1. The clinicopathologic characteristics of the patients with 30 NSCLC participants employed for qRT-PCR assay of PRC1 mRNA expression. **Table S2.** The primers used in qPCR analysis. **Table S3.** The relative information on the antibodies in our study. (DOCX 26 kb)

Additional file 2: Figure S1. The mRNA expression level of PRC1 is highly upregulated in human lung carcinomas and its prognostic significance in the publicly available database. (**A**) PRC1 gene expression is upregulated in lung carcinomas compared to normal lung tissues. Microarray data analyses of PRC1 gene expression in human normal lung and cancer tissues (Hou (ref. [14]), Selamat (ref. [15]), Su (ref. [16]), and Landi (ref. [17] were plotted using the Oncomine software (http://www.oncomine.org). The boxes represent the 25th through 75th percentiles. The horizontal lines represent the medians. The whiskers represent the 10th and 90th percentiles, and the asterisks represent the end of the ranges. (AD, adenocarcinoma; LCC, large cancer lung carcinoma; SCC, squamous carcinoma; ***p ≤ 0.001). B, PRC1 high mRNA expression is associated with poor survival in NSCLC. Kaplan–Meier plots of overall survival (up panel) and progression-free survival (down panel): comparison of patients with high versus low mRNA expression of PRC1 in NSCLC patients stratified by different histological types. The Kaplan-Meier plots were generated using Kaplan-Meier Plotter (http://www.kmplot.com). Patients with high PRC-1 mRNA expression had a worse OS and PFS among NSCLC and lung adenocarcinoma but not in lung squamous carcinoma. (TIFF 3373 kb)

Additional file 3: Figure S2. qRT-PCR analysis of PRC1 expression level in 5 human NSCLC cell lines and three cells transfected lentiviruses shPRC1. (A) Expression of PRC1 was detected by qPCR in NSCLC cell lines (A549, SPC-A1, H1299, H1703, and SK-MES-1). β-actin was used as a loading control. (B) PRC1 expression was confirmed by qPCR in lung adenocarcinoma cells (A549, SPC-A1, and H1299) transduced with lentiviruses expressing scramble (shControl) or PRC1-targeting (shPRC1–1 and shPRC1–2) shRNA. Cells were harvested 5 days after viral transduction. β-actin was used as a loading control. (TIFF 1695 kb)

Additional file 4: Figure S3. PRC1 knockdown significantly attenuates NSCLC cell migration in vitro. Transwell assays were performed to determine the migratory abilities of A549 (A), SPC-A1 (B), and H1299 (C) cells transduced with lentiviruses expressing the indicated shRNA. (TIFF 21391 kb)

Additional file 5: Figure S4. PRC1 knockdown leads to the G2/M phase arrest in vitro*.* Cell-cycle analysis was determined in A549 (A), SPC-A1 (B), and H1299 (C) cells transduced with shPRC1 or shControl. The DNA content was quantified by flow cytometric analysis. (TIFF 4614 kb)

Additional file 6: Figure S5. The morphology of A549 cell transduced with shControl and shPRC1. A549 cells transduced with shPRC1–1(B) and shPRC1–2(C) were shrunken and detached compared to the control group (A). (TIFF 7217 kb)

Additional file 7: Figure S6. PRC1 knockdown leads to apoptosis in vitro*.* Apoptosis in A549 (A) and SPC-A1 (B) cells transduced with shPRC1 was performed by flow cytometry. (TIFF 3598 kb)

## Abbreviations

AD: Adenocarcinoma
CDKs: Cyclin dependent kinases
DEGs: Differentially expressed genes
HSF2: Heat shock transcription factor 2
IHC: I Mmunohistochemistry
KEGG: Kyoto encyclopedia of genes and genomes
MAPs: Microtubule-associated proteins
NGS: Next generation sequencing
NSCLC: Non-small cell lung cancer
PRC1: Protein regulator of cytokinesis-1
SCC: Squamous cell carcinoma
TCF: Transcription factor
TMAs: Tissue microarrays
