# Supplementary material for: PRC1 contributes to tumorigenesis of lung adenocarcinoma in association with the Wnt/β-catenin signaling pathway
Source: Mol Cancer. 2017 Jun 24;16:108. doi: 10.1186/s12943-017-0682-z (PMC5483280; doi:10.1186/s12943-017-0682-z)
Supplement: Supplementary file 1 — The clinicopathologic characteristics of the patients with 30 NSCLC participants employed for qRT-PCR assay of PRC1 mRNA expression. Table S2. The primers used in qPCR analysis. Table S3. The relative information on the antibodies in our study. (DOCX 26 kb) [file 12943_2017_682_MOESM1_ESM.docx]

Table S1 The clinicopathologic characteristics of the patients with 30 NSCLC participants employed for qRT-PCR assay of PRC1 mRNA expression

|  | Number of  Patients |
| --- | --- |
|  |  |
| All patients | 30 |
| Gender |  |
| Male | 18 |
| Female | 12 |
| Age |  |
| <65 y. o. | 13 |
| ≥65 y. o. | 17 |
| Size of tumor |  |
| ≤3cm | 11 |
| >3cm | 19 |
| Grade |  |
| Low | 8 |
| High | 22 |
| Lymph node metastasis (pN) |  |
| N0 | 10 |
| N1-3 | 20 |
| p-TNM stages |  |
| I | 11 |
| II | 7 |
| III | 12 |

Table S2 The primers used in qPCR analysis.

| Primer Name | Primer Sequence 5’-3’ |
| --- | --- |
| PRC1-F | TAGACCACACCCCAGACACA |
| PRC1-R | GTGGCCACAGCTTCTCTTTC |
| shPRC1-1 | CCTGAAGGAAAGACTCATCAA |
| shPRC1-2 | CAGGAACATTCAAAGGCATTT |
| Actin-F | TGACGTGGACATCCGCAAAG |
| Actin-R | CTGGAAGGTGGACAGCGAGG |
| WNT8B-F | AGAAGTACCACGCAGCACTC |
| WNT8B-R | GCAGCCCTAGCGTTTTGTTC |
| cyclin D2-F | CTGGGGAAGTTGAAGTGGAA |
| cyclin D2-R | GAGCAATGAAGGTCTGAGCA |
| VANGL2-F | CTTCATCTCTGTCGCCTTCA |
| VANGL2-R | GGAAAACCAGCACCATAAGC |
| PRKCB-F | AGCCAAAAGCTAGAGACAAGAGA |
| PRKCB-R | GGCTCAACGATGGAGTTTGC |
| TCF7L1-F | CAGTCAAGGACACGAGGTCA |
| TCF7L1-R | GCTGTAGGTGATGAGGGGAGT |
| NFATC4-F | CACCGAGTCACGAATCTCCC |
| NFATC4-R | TTTTCCTCCCCGAACACCAG |
| SFRP4-F | CGAACTCAAGTCCCGCTCAT |
| SFRP4-R | ACTGTTCTCCGCTGTTCCTG |
| PLCB4-F | GAATGCTCCCTCATCAACAG |
| PLCB4-R | ACACAAACTATCCGCCCTTC |
| FZD3-F | GTGAATGAGAGCCGACAGGT |
| FZD3-R | ATGGGTGGATGTTCCTTGAG |
| PRICKLE2-F | AGGCTGTGCTTTGGAAGAGT |
| PRICKLE2-R | TGCTTGATTCGCAGTTTCTC |
| APC-F | GCTCAAACCAAGCGAGAAGT |
| APC-R | AGCATCTGGAAGAACCTGGA |
| DKK1-F | AGCACCTTGGATGGGTATTC |
| DKK1-R | CACACTTGACCTTCTTTCAGGA |
| MYC-F | TTCGGGTAGTGGAAAACCAG |
| MYC-R | CCTCGTCGCAGTAGAAATACG |
| JUN-F | TGACTGCAAAGATGGAAACG |
| JUN-R | CAGGTTCAGGGTCATGCTC |

Table S3 The relative information on the antibodies in our study.

| Antibody | Vender | Cat.No. | Dilution | Host Species | Application |
| --- | --- | --- | --- | --- | --- |
| PRC1 | abcam | 51248 | 1:500 | Rabbit | IHC |
| PRC1 | Santa cruz | 8356 | 1:1000 | Rabbit | WB |
| β-actin | CST | 4970 | 1:1000 | Rabbit | WB |
| Ki-67 | CST | 9449 | 1:400 | Mouse | IHC |
| CyclinD1 | CST | 2922 | 1:1000 | Rabbit | WB |
| P27 | CST | 3686 | 1:1000 | Rabbit | WB |
| P21 | Santa cruz | 397 | 1:1000 | Rabbit | WB |
| Cdc25c | CST | 4688 | 1:1000 | Rabbit | WB |
| Cyclin B1 | CST | 4138 | 1:1000 | Rabbit | WB,IHC |
| CDC2 | CST | 9116 | 1:1000 | Rabbit | WB,IHC |
| PARP | Santa cruz | 7150 | 1:1000 | Rabbit | WB |
| Bcl-xl | CST | 2764 | 1:1000 | Rabbit | WB |
| Bcl-2 | CST | 2870 | 1:1000 | Rabbit | WB |
| Bax | CST | 5023 | 1:1000 | Rabbit | WB |
| Caspase 3 | CST | 9662 | 1:1000 | Rabbit | WB |
| Cleaved Caspase-3 | CST | 9661 | 1:1000 | Rabbit | WB |
| CyclinD2 | CST | 3741 | 1:1000 | Rabbit | WB,IHC |
| Wnt8b | abcam | 66307 | 1:500 | Rabbit | WB |
| TCF3 | CST | 2833 | 1:1000 | Rabbit | WB |
| FZD3 | abcam | 75233 | 1:500 | Rabbit | WB |
| β-catenin  Non-phospho (Active) | CST | 8814 | 1:1000 | Rabbit | WB,IHC |
| c-Myc | Santa cruz | 40 | 1:1000 | Mouse | WB,IHC |
| c-Jun | CST | 9165 | 1:1000 | Rabbit | WB,IHC |

CST: Cell Signaling Technology; WB: Western blotting; IHC: Immunohistochemical staining.
