# Supplementary figures and images for: PRC1 contributes to tumorigenesis of lung adenocarcinoma in association with the Wnt/β-catenin signaling pathway
Source: Mol Cancer. 2017 Jun 24;16:108. doi: 10.1186/s12943-017-0682-z (PMC5483280; doi:10.1186/s12943-017-0682-z)

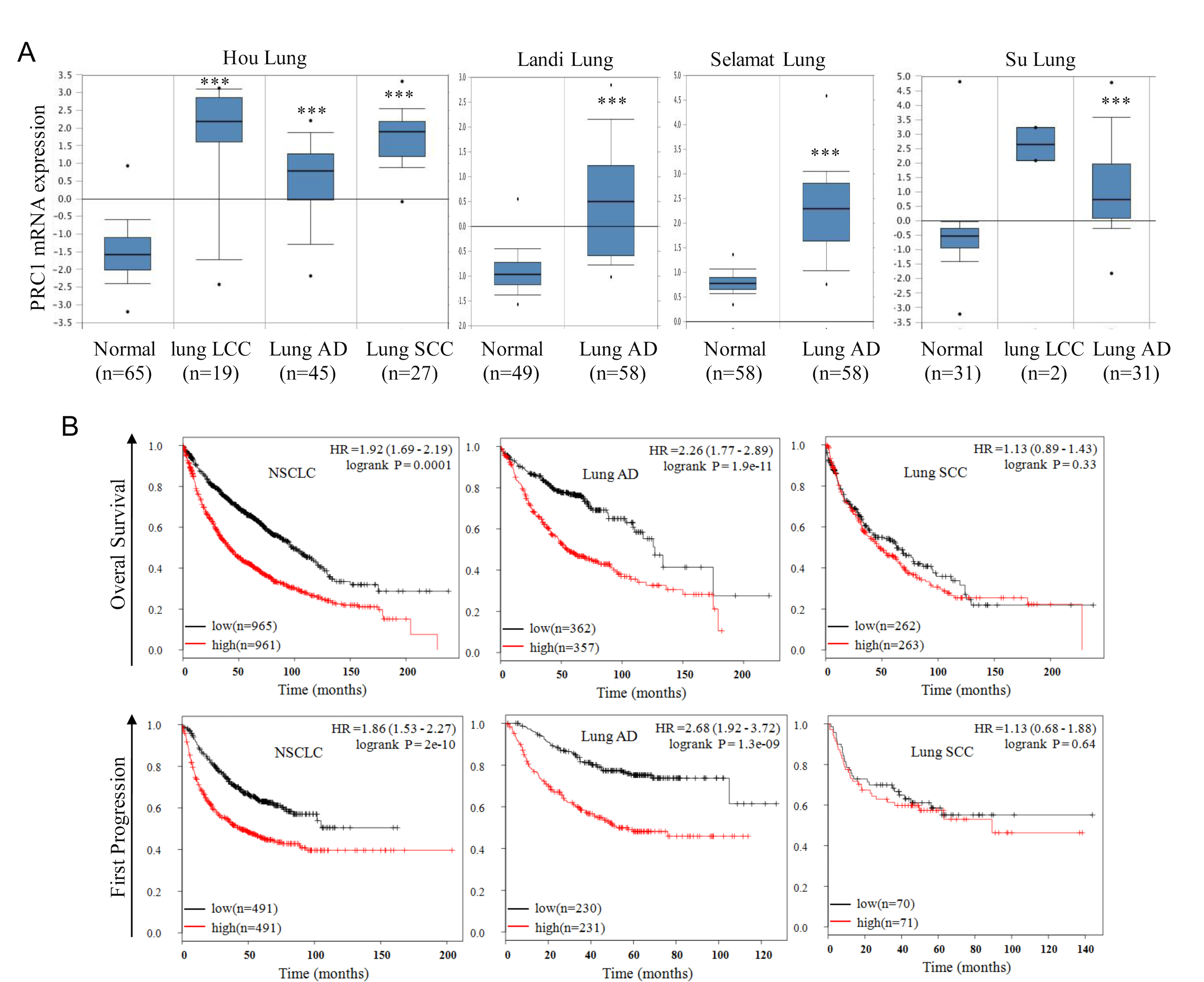

Supplement: Supplementary file 2 — The mRNA expression level of PRC1 is highly upregulated in human lung carcinomas and its prognostic significance in the publicly available database. (A) PRC1 gene expression is upregulated in lung carcinomas compared to normal lung tissues. Microarray data analyses of PRC1 gene expression in human normal lung and cancer tissues (Hou (ref. [14]), Selamat (ref. [15]), Su (ref. [16]), and Landi (ref. [17] were plotted using the Oncomine software (http://www.oncomine.org). The boxes represent the 25th through 75th percentiles. The horizontal lines represent the medians. The whiskers represent the 10th and 90th percentiles, and the asterisks represent the end of the ranges. (AD, adenocarcinoma; LCC, large cancer lung carcinoma; SCC, squamous carcinoma; ***p ≤ 0.001). B, PRC1 high mRNA expression is associated with poor survival in NSCLC. Kaplan–Meier plots of overall survival (up panel) and progression-free survival (down panel): comparison of patients with high versus low mRNA expression of PRC1 in NSCLC patients stratified by different histological types. The Kaplan-Meier plots were generated using Kaplan-Meier Plotter (http://www.kmplot.com). Patients with high PRC-1 mRNA expression had a worse OS and PFS among NSCLC and lung adenocarcinoma but not in lung squamous carcinoma. (TIFF 3373 kb) [file 12943_2017_682_MOESM2_ESM.tif]

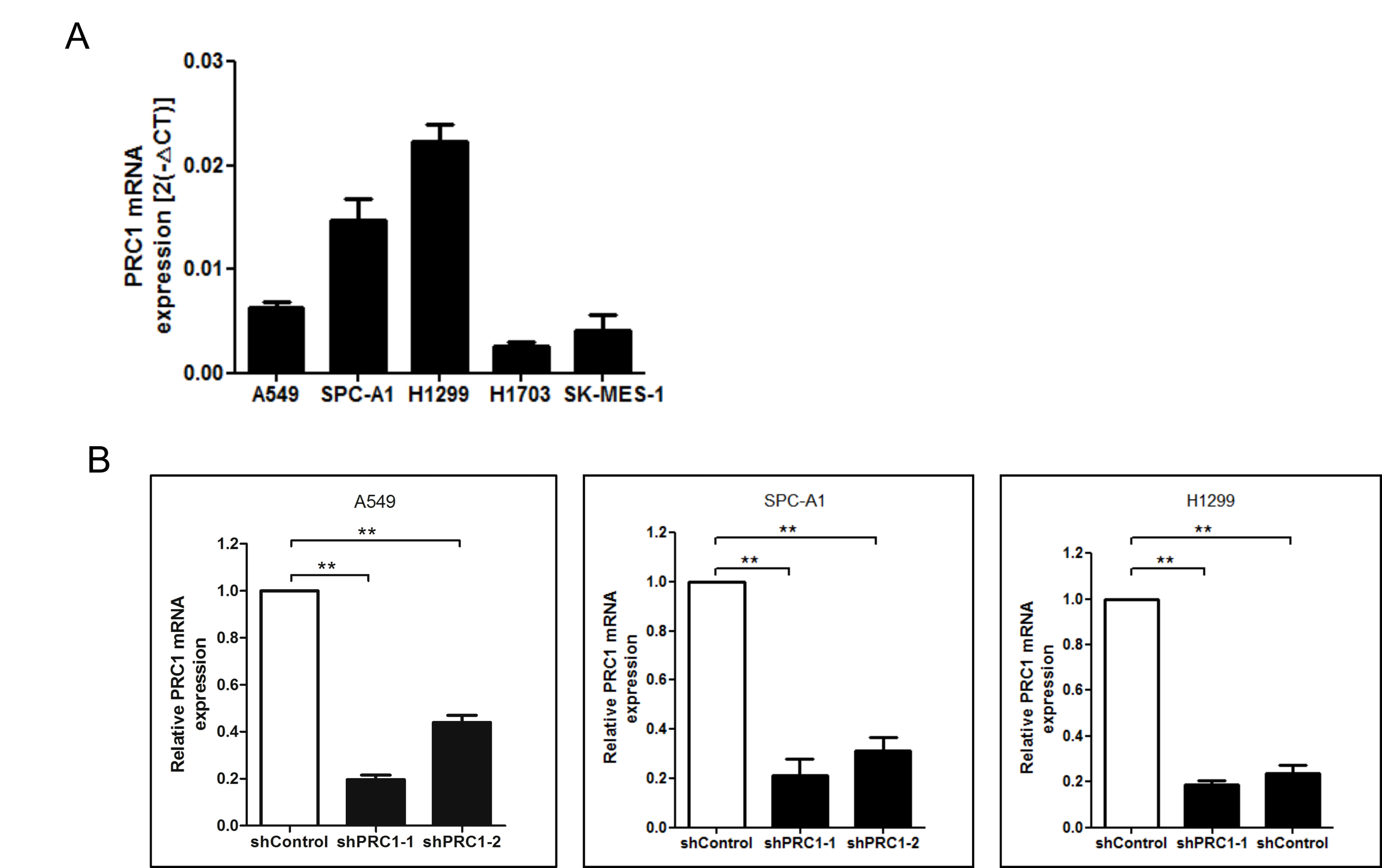

Supplement: Supplementary file 3 — qRT-PCR analysis of PRC1 expression level in 5 human NSCLC cell lines and three cells transfected lentiviruses shPRC1. (A) Expression of PRC1 was detected by qPCR in NSCLC cell lines (A549, SPC-A1, H1299, H1703, and SK-MES-1). β-actin was used as a loading control. (B) PRC1 expression was confirmed by qPCR in lung adenocarcinoma cells (A549, SPC-A1, and H1299) transduced with lentiviruses expressing scramble (shControl) or PRC1-targeting (shPRC1–1 and shPRC1–2) shRNA. Cells were harvested 5 days after viral transduction. β-actin was used as a loading control. (TIFF 1695 kb) [file 12943_2017_682_MOESM3_ESM.tif]

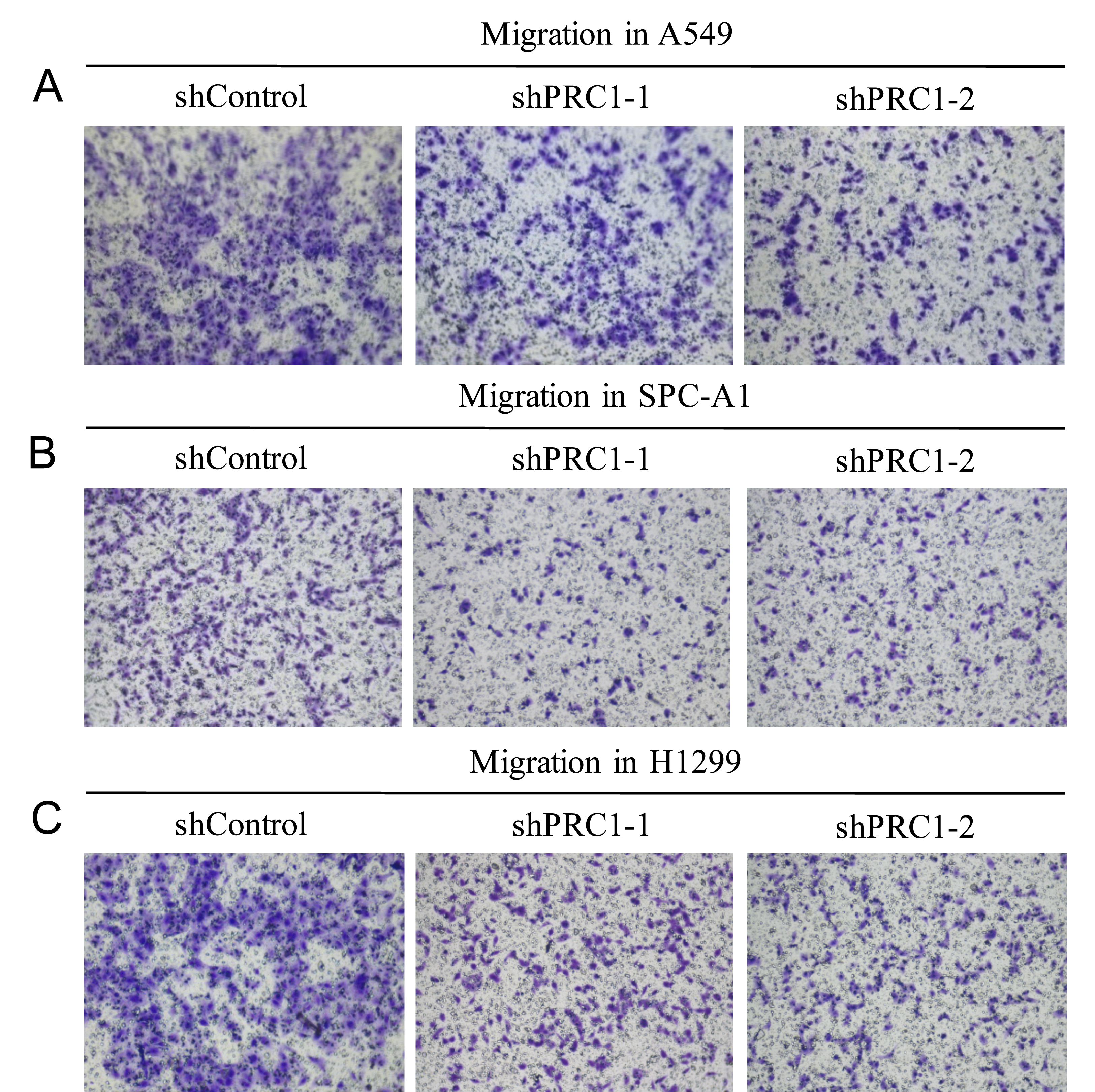

Supplement: Supplementary file 4 — PRC1 knockdown significantly attenuates NSCLC cell migration in vitro. Transwell assays were performed to determine the migratory abilities of A549 (A), SPC-A1 (B), and H1299 (C) cells transduced with lentiviruses expressing the indicated shRNA. (TIFF 21391 kb) [file 12943_2017_682_MOESM4_ESM.tif]

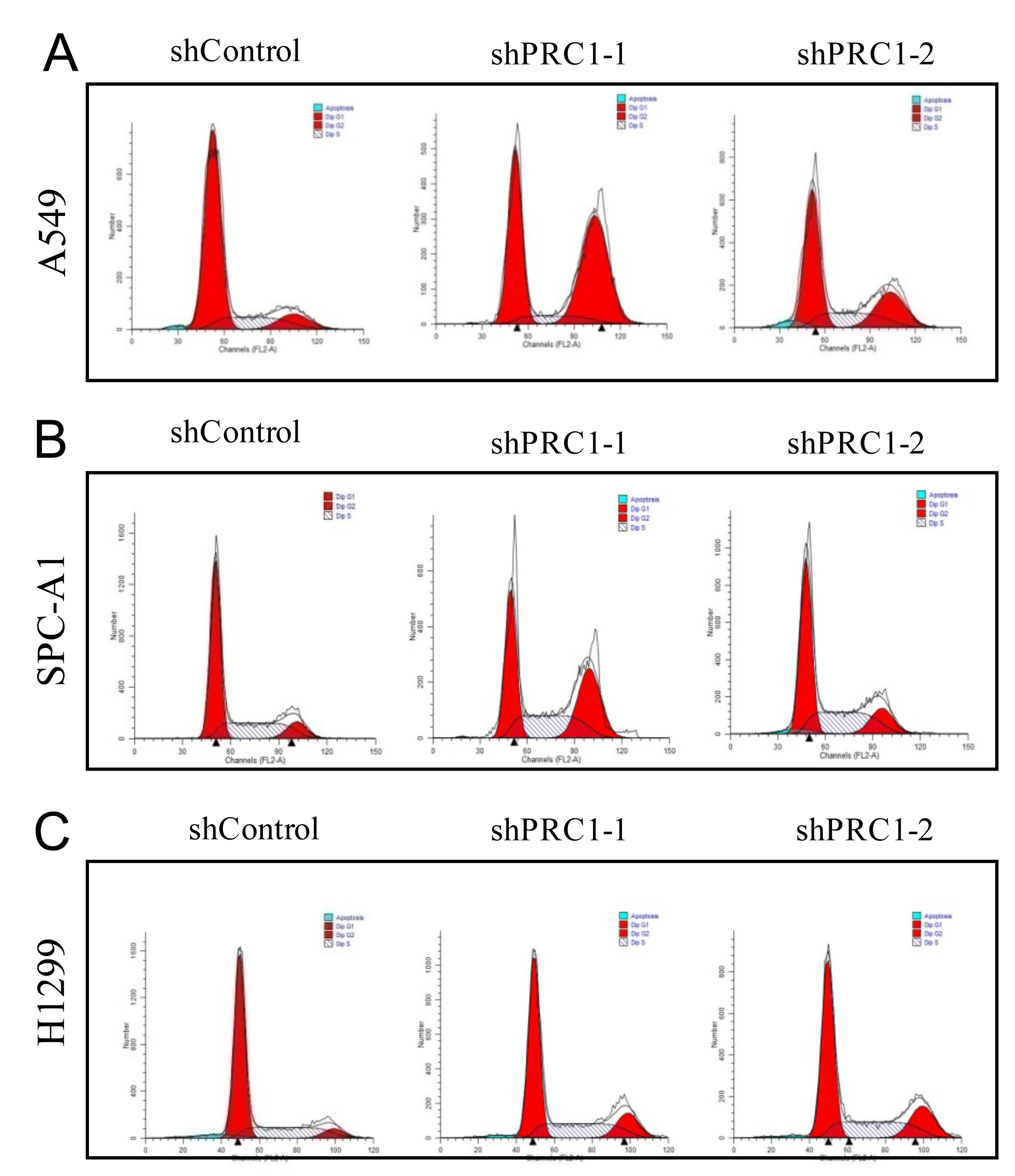

Supplement: Supplementary file 5 — PRC1 knockdown leads to the G2/M phase arrest in vitro. Cell-cycle analysis was determined in A549 (A), SPC-A1 (B), and H1299 (C) cells transduced with shPRC1 or shControl. The DNA content was quantified by flow cytometric analysis. (TIFF 4614 kb) [file 12943_2017_682_MOESM5_ESM.tif]

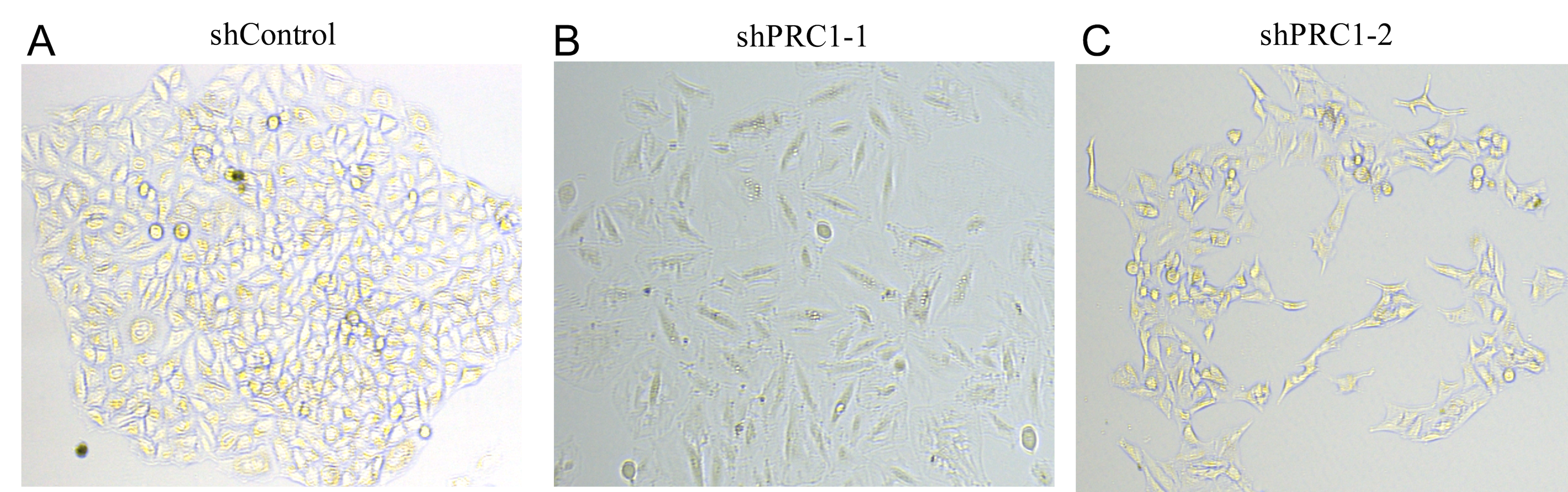

Supplement: Supplementary file 6 — The morphology of A549 cell transduced with shControl and shPRC1. A549 cells transduced with shPRC1–1(B) and shPRC1–2(C) were shrunken and detached compared to the control group (A). (TIFF 7217 kb) [file 12943_2017_682_MOESM6_ESM.tif]

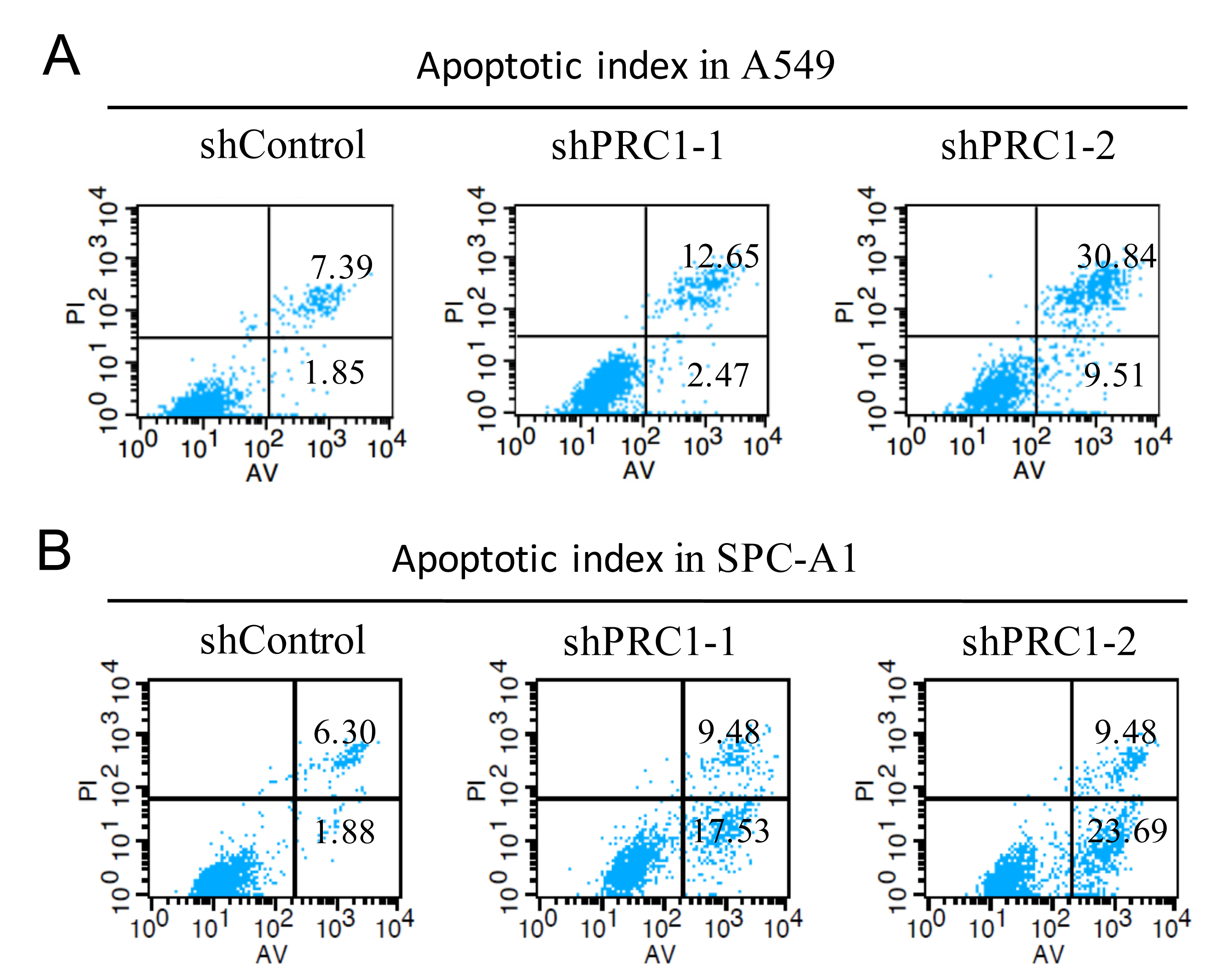

Supplement: Supplementary file 7 — PRC1 knockdown leads to apoptosis in vitro. Apoptosis in A549 (A) and SPC-A1 (B) cells transduced with shPRC1 was performed by flow cytometry. (TIFF 3598 kb) [file 12943_2017_682_MOESM7_ESM.tif]
